# Where and why malaria persists in heterogeneous transmission settings: insights from routine health facility data

**DOI:** 10.1136/bmjgh-2025-023003

**Published:** 2026-08-07

**Authors:** Camille Morlighem, Corentin Visée, Chibuzor Christopher Nnanatu, Mame Wodji Tine, Aminata Niang Diène, Catherine Linard

**Affiliations:** 1 Sorbonne Public Health Institute (SPH), NEMESIS, Sorbonne Université, INSERM, F75012, Paris, France; 2 Department of Geography, University of Namur, 5000, Namur, Belgium; 3 ILEE, University of Namur, 5000, Namur, Belgium; 4 WorldPop, School of Geography and Environmental Science, University of Southampton, Southampton, SO17 1BJ, UK; 5 Department of Statistics, Nnamdi Azikiwe University, PMB 5025, Awka, Nigeria; 6 Department of Geography, University Cheikh Anta Diop of Dakar (UCAD), Dakar, Senegal

**Keywords:** Malaria, Health Services Accessibility, Incidence

## Abstract

**Background:**

Malaria distribution in sub-Saharan Africa is becoming increasingly spatially heterogeneous. Pre-elimination areas, high-transmission settings and urban areas with increasing transmission may all coexist within the same country. Effective intervention planning requires identifying high-risk areas and their drivers—an area in which geospatial models can provide valuable insights. Yet these models often assume stationary risk factors across transmission levels and frequently rely on prevalence survey data, which are unreliable in low-transmission settings. This study uses routine public-sector health facility data (ie, DHIS2) to model malaria incidence in Senegal from 2017 to 2021, accounting for transmission levels, and introduces novel fine-scale estimates of reported malaria incidence derived from dasymetric disaggregation.

**Methods:**

Health facility catchment populations were estimated using a probabilistic approach, allowing individuals to attend multiple facilities based on relative travel time. Malaria incidence per catchment was modelled using high-resolution environmental and socioeconomic covariates within a Bayesian hierarchical modelling framework. Separate models were fitted by season and endemicity-urbanisation level to assess the heterogeneity of risk factors. We adapted dasymetric disaggregation methods from population mapping to redistribute facility-level cases to 1 km pixels.

**Results:**

Estimated malaria incidence increased from 60 cases per 1000 people in 2017 to 71 cases in 2021. Key risk factors varied across endemicity-urbanisation levels; some factors were uniquely associated with low (eg, distance to roads), moderate (eg, cropland) and high-transmission settings (eg, flooded vegetation). Fine-scale maps represent malaria cases that the public-sector health system routinely reports, rather than the total malaria burden, and revealed potential underdiagnosis in remote areas.

**Conclusions:**

Our findings emphasise the importance of considering endemicity levels when evaluating malaria risk factors in heterogeneous transmission contexts. As health system data quality continues to improve, this approach offers a scalable alternative for mapping reported malaria incidence, supporting better targeted interventions and the identification of underdiagnosed areas.

**WHAT IS ALREADY KNOWN ON THIS TOPIC**

- Although malaria transmission is becoming increasingly spatially heterogeneous across sub-Saharan African countries, malaria risk models often assume stationary risk factors and depend on prevalence survey data, which are less reliable in low-transmission settings.

**WHAT THIS STUDY ADDS**

- Malaria risk factors vary by transmission and urbanisation level, with some factors showing contrasting effects across contexts.
- Dasymetric disaggregation enables the production of high-resolution (eg, 1 km) estimates of reported malaria cases.
- Fine-scale maps show higher reported malaria incidence in urban areas, highlighting potential underdiagnosis in remote areas.

**HOW THIS STUDY MIGHT AFFECT RESEARCH, PRACTICE OR POLICY**

- Our findings emphasise the need to analyse malaria risk factors within specific endemicity contexts, as national models may mask local heterogeneity.
- With the growing use of District Health Information Software (DHIS2) in sub-Saharan Africa, this study provides a scalable framework for producing fine-scale incidence maps from routine health data, thereby facilitating the development of more effective intervention strategies.

## Introduction

 Despite progress over the past decade, global malaria mortality and morbidity rates are stalling.^1^ The distribution of malaria in sub-Saharan Africa (SSA) is becoming increasingly spatially heterogeneous due to factors such as intensified intervention strategies, climate change and urbanisation. Pre-elimination areas,^2–5^ high-transmission settings and rapidly urbanising areas with increasing transmission^6–9^ may all coexist within the same country. Designing effective, context-specific interventions requires identifying areas at risk across this spectrum and the contextual drivers behind them. Geospatial models can provide key insights in this regard.

However, previous malaria modelling studies (eg, in Ghana,^10^ Rwanda,^11^ Nigeria,^12 13^ Mozambique^14^ and Senegal^15^) have often assumed spatial stationarity in determinants and their relationships with malaria.^16^ Yet heterogeneous transmission settings often reflect distinct social, environmental and epidemiological contexts, such that these assumptions may not hold. Endemicity directly influences immunity, meaning adults in low versus high-endemic areas have different levels of vulnerability,^17^ and risk factors may differ accordingly (eg, migration may pose a greater risk in low-endemic areas). More generally, relationships with vector-borne diseases may vary by vector species,^18^ season,^19^ spatial scale of analysis (from household to regional level)^18 20 21^ and urban–rural setting.^22^ In addition, control versus elimination interventions and their effects vary according to the level of transmission.^23^ Accounting for endemicity is therefore essential when analysing risk factors, especially as regional differences in endemicity are likely to become even more pronounced in the future.

Fine-scale malaria models are often based on household surveys testing for malaria in children under 5, such as the Demographic and Health Surveys (DHS) and Malaria Indicator Surveys. However, their malaria prevalence estimates do not account for high-risk adolescents^24 25^ and are unreliable in low-transmission settings due to sampling zeros.^26 27^ Prevalence survey data may also become scarcer following the closure of the United States Agency for International Development (USAID) in 2025.^28^ Alternatively, routine case data collected monthly by health facilities can be used to estimate malaria incidence continuously over time and across large spatial scales.^29^ Although these data are affected by under-reporting and care-seeking gaps,^26 29^ they capture the burden across all age groups.^29^ Given the growing demand for disease estimates at the locality or health facility (HF) level,^30^ recent studies have investigated the use of such data for spatiotemporal modelling and fine-scale prediction of malaria incidence.^3 31–33^ In this study, we propose using dasymetric disaggregation, a method commonly used to disaggregate population census estimates,^34 35^ to produce fine-scale maps of reported malaria incidence.

This study aims to model malaria incidence while accounting for transmission intensity, using routine District Health Information Software (DHIS2) data at HF level, and high-resolution environmental and socioeconomic variables. Senegal is used as a case study for 2017–2021, given its highly heterogeneous transmission settings. Catchment areas are delineated by allowing individuals to attend multiple health facilities depending on travel time, rather than assuming non-overlapping catchments. Spatiotemporal models of malaria incidence are built within a Bayesian hierarchical framework, with separate models fitted by season and endemicity level to evaluate variations in risk factors. Finally, dasymetric disaggregation is applied to generate high-resolution (1 km) maps of reported malaria incidence to support targeted intervention planning.

## Methods

### Case study

**Figure 1:**
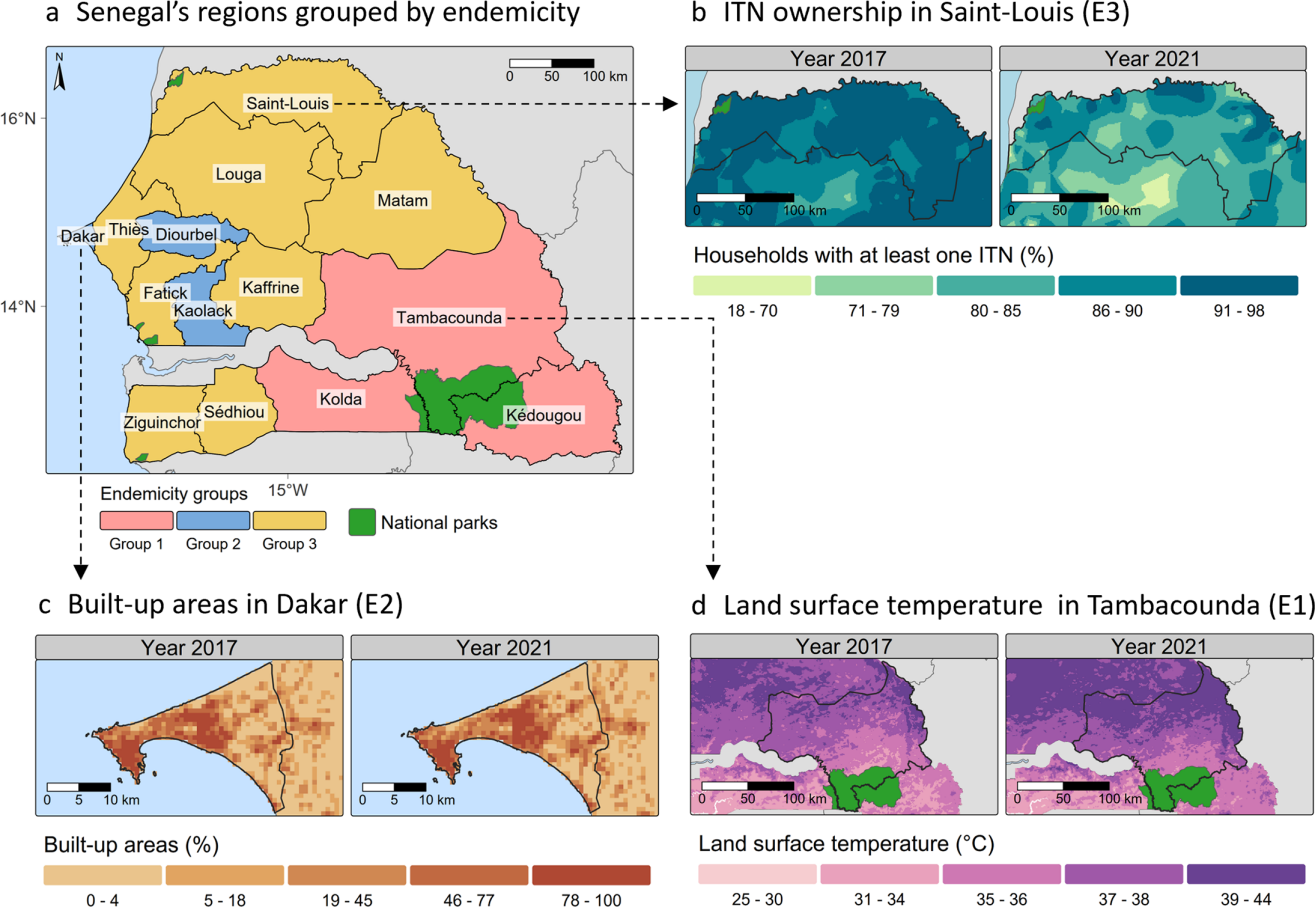
(**a**) Endemicity groups and (b–d) spatiotemporal patterns of selected covariates across Senegal. In (**a**), regions are grouped as follows: group 1—rural south-eastern regions with high transmission (65% of malaria cases); group 2—western urban regions (30% of cases); and group 3—northern and south-western regions with mixed urbanisation (5% of cases). (b–d) show geospatial covariates in 2017 and 2021: insecticide-treated net (ITN) ownership in Saint-Louis, built-up areas in Dakar and land surface temperature in Tambacounda, respectively.

Despite significant efforts in recent decades and a 2030 elimination target, malaria cases have stagnated and increased in Senegal since 2019,^36^ resulting in an estimated 1 199 388 cases and 3070 deaths in 2023.^1^ Senegal has a heterogeneous malaria epidemiological profile, with three major endemicity groups and distinct levels of urbanisation (figure 1a)^36^: (1) rural south-eastern regions (Kolda, Kédougou and Tambacounda; KKT area) with historically high transmission, accounting for 12% of the population but 65% of malaria cases; (2) western urban regions (Dakar, Diourbel and Kaolack) with moderate transmission and 30% of cases; and (3) northern and south-western regions, with both poorly and highly urbanised areas,^37^ low to very low transmission and pre-elimination areas.^36^

### Routine malaria data

Routine malaria data (2017–2021) were extracted from the DHIS2 by the Laboratoire Dynamiques Territoriales et Santé at Université Cheikh Anta Diop de Dakar (Senegal). Annual reporting rates remained consistently high (97%)^38 39^ over this period but declined from 2022 onwards (eg, 27% in 2023) due to health worker strikes, making data unreliable for analysis.^40^ Data consist of malaria cases in all age groups, confirmed by rapid diagnostic tests (RDTs), per month and per HF. Only public facilities were included^5 31^ due to low treatment-seeking rate (10%) in the private sector^41^ and poor private sector DHIS2 reporting.^38^

**Figure 2:**
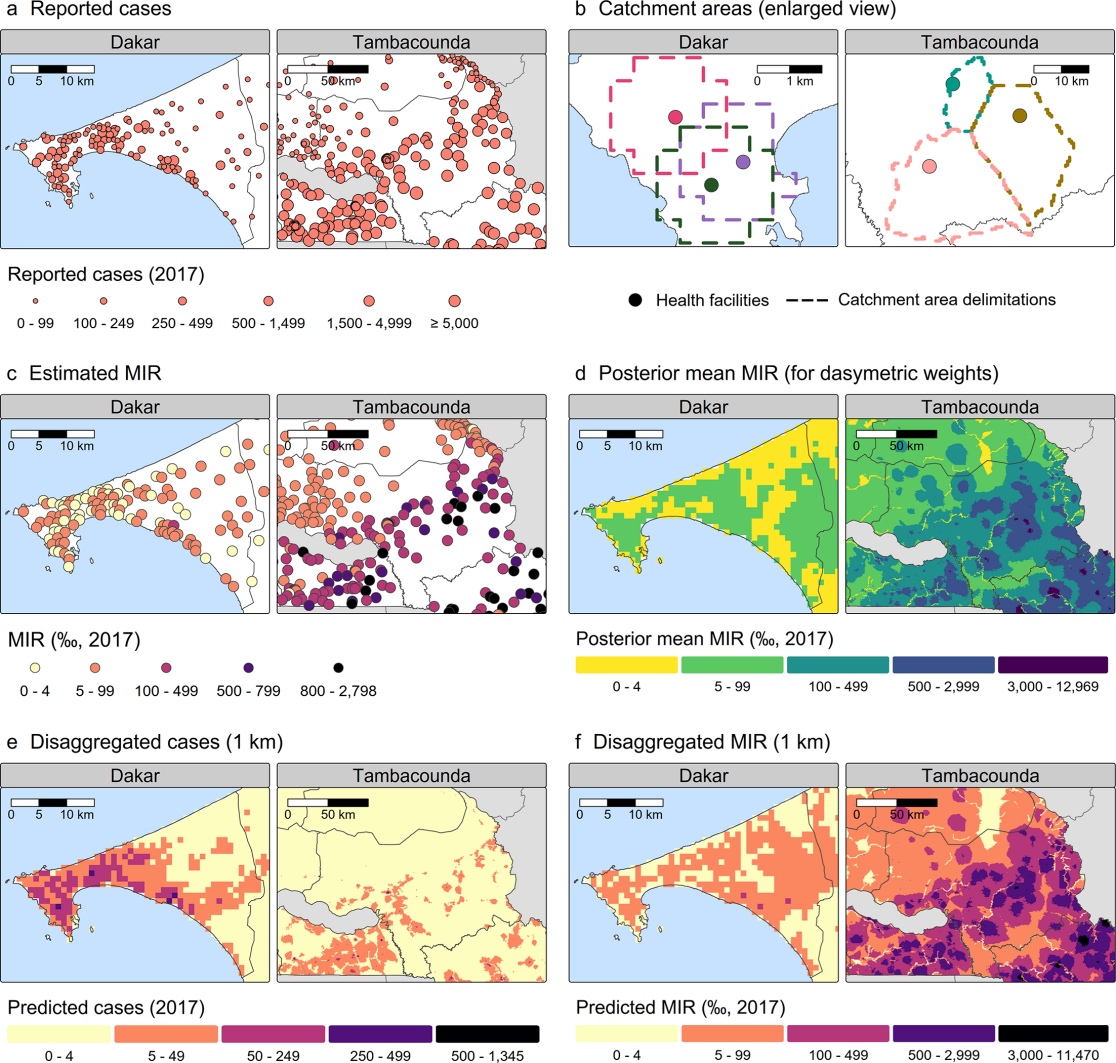
Intermediate and final steps of the fine-scale reported incidence mapping workflow, with examples from Dakar and Tambacounda (2017). After geolocating (**a**) health-facility reported cases, (**b**) travel-based catchment areas are generated; the enlarged view contrasts overlapping catchments in dense facility settings (Dakar) with non-overlapping catchments in sparse settings (Tambacounda). (**c**) Malaria incidence rates (MIR, per 1000 people) are estimated using catchment populations and modelled. Bayesian models produce (**d**) 1 km posterior mean MIR, which are used to derive dasymetric weights. These weights enable (**e**) cases to be redistributed at a fine spatial scale, and dividing the redistributed counts by the treatment-seeking population yields (**f**) fine-scale MIR.

Public health facilities missing case data for the entire 2017–2021 period (8%) were excluded because such gaps could indicate under-reporting, non-functional facilities or lack of diagnostic capacity.^38 42^ Facilities were geolocated using the database of Gueye *et al*,^43^ which consolidated georeferenced HF data for Senegal. Facilities not present in this database were verified using OpenStreetMap and Google Maps. Through this process, 90% of public facilities (n=1472) with at least one reported case were geolocated (online supplemental figures S1 and S2 and online supplemental table S1). For these facilities, gaps in reported case counts were inferred during modelling. The methodological workflow for deriving fine-scale incidence estimates from reported cases is summarised in figure 2, with examples from Dakar and Tambacounda.

### Geospatial covariates

Morlighem *et al*^44^ published a conceptual framework of malaria risk as influenced by environmental hazard and societal vulnerability and mapped it to open-access geospatial covariates. Covariates in our study (online supplemental table S2) were selected based on this work and included (1) a set of gridded environmental and socioeconomic covariates (2017–2021, except for time-invariant covariates) and (2) DHS indicators (2017–2021) influencing vulnerability (eg, stunting in children, insecticide-treated net (ITN) ownership and the proportion of Fula people, who have lower genetic susceptibility to malaria and include migratory groups in Senegal). We created continuous surfaces of these indicators following previous work^44 45^ (see online supplemental file 1 and online supplemental figures S3–S7). Covariates were resampled to a 1×1 km grid to ensure spatial alignment. Examples of selected covariates are shown in figure 1b–d.

### Catchment population

#### Computation of travel time

People were assumed to walk to health facilities, as walking is the predominant mode of travel for seeking treatment for fever in SSA,^46^ including in Senegal.^47^ Besides, walking time to health facilities has been identified as a key malaria risk factor in Senegal.^44^ Walking time was calculated considering slope, land cover, roads and rivers (online supplemental table S3) adapting Alegana *et al*^48^ (see online supplemental file 1).

#### Treatment-seeking population

Unconstrained gridded population counts (100 m, resampled to 1 km) were extracted from the WorldPop project for 2017–2020^49 50^ and, due to data unavailability at the time of the study, projected to 2021 by fitting a simple exponential growth model.

In Senegal, 48% of people do not seek care when their child has a fever.^41^ To account for this, we used all available Senegal DHS data (2017–2023) on treatment-seeking at public health facilities among febrile children. Treatment-seeking was modelled as a function of travel time using a spatiotemporal Bayesian logistic regression (see online supplemental file 1). The fitted model was used to generate annual probability surfaces of treatment-seeking for 2017–2021 (online supplemental figure S8). The treatment-seeking population was calculated by multiplying the population counts by these probabilities^3 48^ and by a regional case geolocation rate (ie, the proportion of geolocated cases) to account for facilities that could not be geolocated (online supplemental table S1).

#### Overlapping catchment areas

The catchment population of a HF refers to the group of people who attend that facility for treatment of fever, while the ‘total *potential* catchment population’ is the group that would attend if 100% of the population sought care. To avoid biases from fixed non-overlapping catchments (eg, unrealistic assumptions and underestimated urban catchment populations),^42 51 52^ health facilities were assigned overlapping catchment areas, assuming that individuals may choose among multiple health facilities depending on relative travel time.

Cases from facilities located in the same 1×1 km pixel were summed.^5 42^ The probability $p\left({\text{pixel}}_{i}\to {\text{HF}}_{j}\right)$ that a treatment-seeking individual in populated pixel i would attend HF j was calculated following^31^:


(1) $$p\left({\text{pixel}}_{i}\to {\text{HF}}_{j}\right)=\frac{d{\left({\text{pixel}}_{i}\to {\text{HF}}_{j}\right)}^{-2}}{\sum_{j=1}^{N_{\text{HF}}}d{\left({\text{pixel}}_{i}\to {\text{HF}}_{j}\right)}^{-2}}$$

where $d\left({\text{pixel}}_{i}\to {\text{HF}}_{j}\right)$ is the walking time from pixel i to facility j and $N_{\text{HF}}$ is the number of health facilities. Probabilities per pixel sum to 1. As this approach assigns a non-zero probability of attending facilities that are realistically out of reach, we improved it by assuming that individuals would visit any facility within 30 minutes with probabilities computed using equation 1, or would attend the nearest facility when walking time was greater than 30 minutes (ie, with probability 1). This threshold was chosen because 75% of people live at less than 30 minutes from the nearest HF.^47^ The catchment population $E_{j}$ of HF j was calculated as follows:


$$pop_{i\to j}={\text{treatment-seeking population}}_{i}\times p\left({\text{pixel}}_{i}\to {\text{HF}}_{j}\right)$$

(2) $$E_{j}=\sum_{i=1}^{N_{\text{pixel}}}pop_{i\to j}$$

where ${pop}_{i\to j}$ is the population in pixel i attending facility j, and $N_{\text{pixel}}$ is the number of populated pixels with a non-zero probability of attending facility j. Annual catchment areas were generated (2017–2021) using annually updated travel time. For each year t, we considered all facilities that reported cases in year t and/or in any prior year. Figure 2b illustrates an example of catchment areas delineation.

#### Covariate extraction

For land use and land cover, we extracted the proportion of each class per catchment. For other covariates, we extracted the mean values in the catchment area after masking out water pixels. Covariates were standardised using z-score transformation.

### Incidence model

#### Model structure

The malaria incidence rate (MIR) $R_{jt}$ at HF j(j=1,…,N) in year t(t=1,…,5; 2017–2021)—as illustrated in figure 2c—was calculated as follows:


(3) $$R_{jt}=\frac{c_{jt}}{E_{jt}}$$

where $c_{jt}$ is the number of cases reported at facility j in year t and $E_{jt}$ is its catchment population. MIR values are highly right-skewed (online supplemental figure S9), with many near-zero values and a few above 1 due to repeated malaria episodes^53^ and migration.^54^ The log-transformed MIR follows a normal distribution:


$$\log\left(R_{jt}\right)\sim N(\mu_{jt},\sigma_{e}^{2})$$

(4) $$\mu_{jt}=\beta_{0}+X_{jt}^{T}\beta +\xi_{jt}$$

where $\sigma_{e}^{2}$ is the variance of the measurement error, *β*_0_ is the intercept and *X_jt_* is the vector of covariates with regression coefficients *β*. The measurement error is modelled as a zero-mean Gaussian error term representing uncorrelated variations. *ξ_jt_* is a spatiotemporal random effect with first-order autoregressive dynamics and spatially correlated random variations^55^:


(5) $$\xi_{jt}=a\xi_{jt-1}+w_{jt}$$

where $\xi_{j1}\sim N(0,\frac{\sigma_{w}^{2}}{1-a^{2}})$ with autoregressive coefficient |a|§amp;lt;1 and $\sigma_{w}^{2}$ is the marginal variance of the Gaussian field $w_{jt}$. $w_{jt}$ is temporally independent with the following spatiotemporal covariance function^55^:


(6) $$\mathrm{Cov}(w_{jt},w_{kt^{\prime }})=\left\{ \begin{array}{ll} 0, & \text{if }t\neq t^{\prime },\\ \mathrm{Cov}(w_{j},w_{k}), & \text{if }t=t^{\prime }. \end{array}\right.$$

where $\text{Cov}(w_{j},w_{k})$ is the Matérn covariance function (Equation S2, online supplemental file 1). Prior specifications are detailed in online supplemental file 1.

MIR was modelled within a Bayesian hierarchical framework implemented in *R-INLA* with the integrated nested Laplace approximation (INLA)^56^ and the stochastic partial differential equation (SPDE) approach.^57^ Six models were fitted to account for seasonality and endemicity (online supplemental table S4): (1) a benchmark model (BM) with all cases from all health facilities, (2) a wet season model (WM) including cases from all health facilities recorded during the wet season (June–October), (3) a dry season model (DM) including cases from all health facilities recorded during the dry season (November–May), and (4–6) three endemicity models (E1–E3), each restricted to cases from facilities located within the corresponding endemicity group (figure 1a).

Forward stepwise variable selection was implemented with *INLAstep* (*INLAutils* package),^44^ including the spatially and temporally correlated random effects as separate terms due to computational constraints. Multicollinearity was checked by ensuring variance inflation factor (VIF) values <5 for selected covariates.^58^ Models were validated using a 5-repeated fivefold random cross-validation (randomly partitioning health facilities into five folds per repeat; each fold held out once) and temporal block cross-validation (holding out 1 year at a time). Performance was computed on held-out data using the mean absolute error (MAE), root mean square error (RMSE), coefficient of determination (R²) and squared correlation between predicted and observed values (r²).

The models enabled inference of MIR for facilities with missing case data.^3 42^ District-level and national-level estimates were then calculated by multiplying facility-specific MIR (observed, or modelled when missing) by the total *potential* catchment population, aggregating these case counts by district (or nationally) and dividing by the corresponding district (or national) population.

#### Dasymetric disaggregation

We created continuous surfaces of reported malaria cases and incidence rates for 2017–2021 using a dasymetric disaggregation method introduced by Stevens *et al*^34^ for population mapping. In their method, a predicted population-density surface is used as a weighting layer to redistribute census counts from administrative units to pixels. We applied the same principle to redistribute reported malaria cases from health facilities to 1 km pixels.

First, we generated a 1 km MIR surface (figure 2d) using Bayesian geostatistical models (BM, DM, WM). As these models are trained at the catchment level, the resulting MIR surface cannot be interpreted as absolute incidence, but rather as a relative risk surface. For each facility, pixels with a higher predicted malaria risk and a larger attending population are assumed to contribute more to that facility’s total case count.

For facility j, the weight assigned to pixel i in its catchment area is:


(7) $$w_{i\to j}=\frac{R_{i}\times pop_{i\to j}}{\sum_{i=1}^{N_{\text{pixel}}}\left(R_{i}\times pop_{i\to j}\right)}$$

where $R_{i}$ is the MIR in pixel i, ${pop}_{i\to j}$ is the population in pixel i attending facility j (from equation 2) and $N_{\text{pixel}}$ is the number of pixels in its catchment area. Weights sum to 1 over $N_{\text{pixel}}$, ensuring that the sum of the disaggregated cases per catchment matches the facility’s reported total $c_{j}$. The reported cases at the HF level are then redistributed across pixels using these weights. Cases from facility j assigned to pixel i are:


(8) $$c_{i\to j}=w_{i\to j}\times c_{j}$$

The total number of cases in pixel i (figure 2e) is the sum of the contributions from all facilities (n = $N_{\text{HF}}$) whose catchments include pixel i:


(9) $$c_{i}=\sum_{j=1}^{N_{\text{HF}}}c_{i\to j}$$

When $c_{j}$ was missing, we used the exponentiated Bayesian posterior mean log MIR multiplied by the catchment population to distribute cases across pixels. The final 1 km incidence surface (figure 2f) was obtained by dividing the disaggregated case surface (figure 2e) by the pixel treatment-seeking population. We tested two variants for calculating the dasymetric weights (equation 7), using either treatment-seeking populations (${pop}_{i\to j}$), which were calculated from WorldPop constrained estimates (where population is only predicted in settled areas), or unconstrained estimates.^59^

For consistency, we verified that the sum of the disaggregated cases within each region corresponded closely to the reported regional totals. As an external plausibility check, we examined the correlation between fine-scale incidence from the 2017 wet season and malaria prevalence in children aged 0–5 years using data from the 2017 Senegal DHS (mostly conducted during the rainy season). While DHS prevalence and reported clinical incidence capture different epidemiological quantities (influenced by factors such as immunity and healthcare access and reflecting different age groups), a positive relationship between the two would provide supporting evidence for the spatial patterns identified. Cluster-level prevalence was extracted from the DHS, while 1 km reported incidence estimates for the 2017 wet season were averaged within 5 km and 2 km buffers around rural and urban clusters,^60^ respectively.

## Results

### Malaria incidence estimates

MIR calculated after catchment area extraction showed clear spatiotemporal gradients, ranging from 0 to 5323 cases per 1000 population, with higher values concentrated in the southeast (online supplemental figures S11 and S12). Bayesian geostatistical models were used to infer MIR for facilities with missing data (online supplemental figure S13), resulting in 4510 additional cases from 2017 to 2021. Facility-level MIR (observed or modelled where unavailable) were then multiplied by each facility’s total *potential* catchment population. These estimated case counts were summed to produce national and district-level estimates by dividing them by the corresponding (national or district) population.

**Figure 3:**
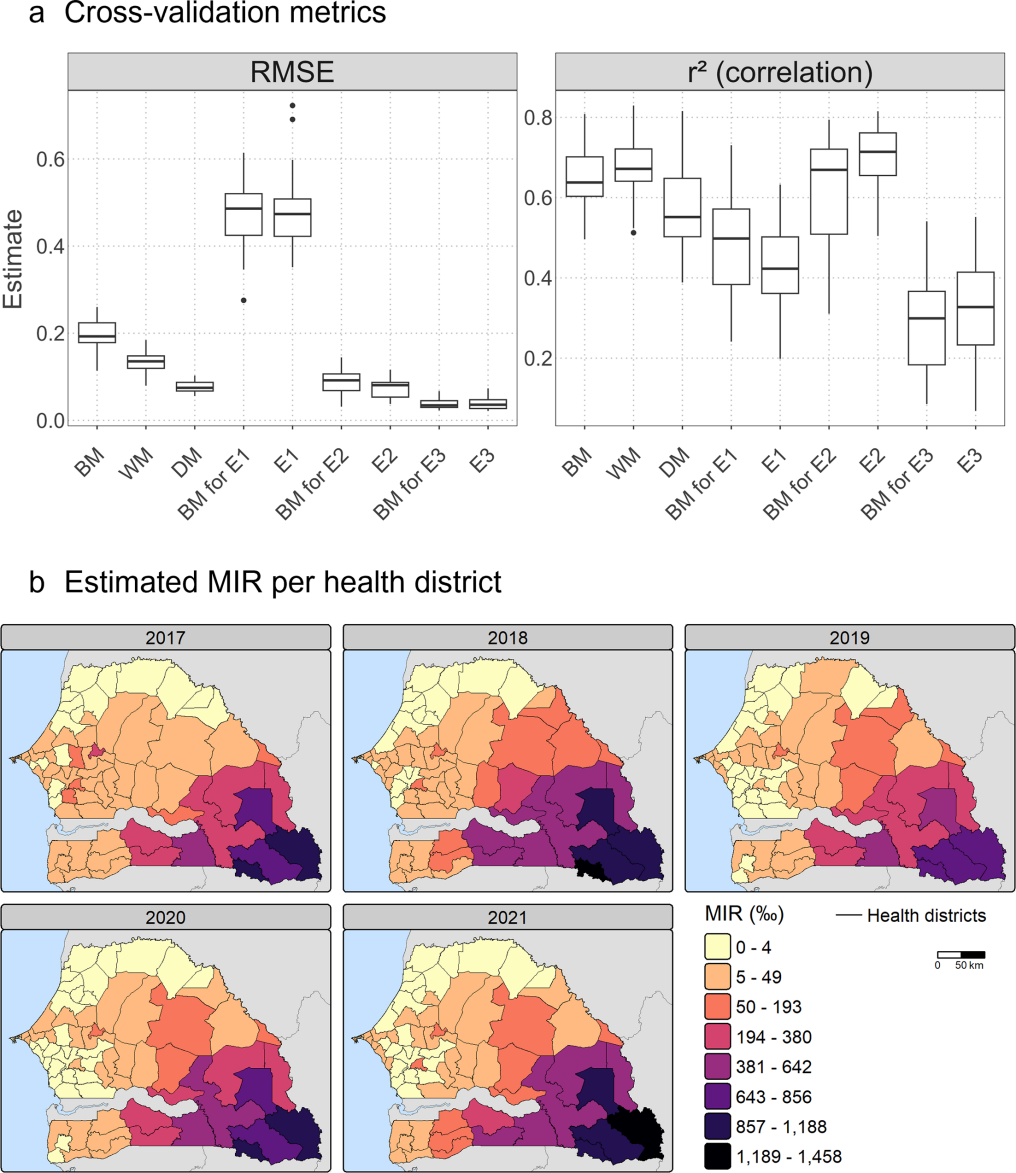
(**a**) RMSE and r^2^ from a 5-repeated fivefold random cross-validation of Bayesian incidence models and (**b**) estimated malaria incidence rates (MIR, per 1000 people) for 2017–2021 per health district. In (**a**), r² is the squared correlation between observed and predicted MIR. Medians are shown as horizontal lines and dots mark outliers. In (**b**), facility-level case counts were obtained by multiplying the health facility’s MIR (or, if unreported, the posterior mean MIR from BM) by the total *potential* catchment population. District-level MIR were computed by summing facility cases and dividing by district population. MIR above 1000‰ reflect malaria repeat episodes^53^ and migration.^54^ BM, benchmark model; DM, dry season model; RMSE, root mean square error; WM, wet season model.

At the national level, the annual MIR increased from 59.75 cases per 1000 population in 2017 to 75.91 in 2018, decreased to 48.2 in 2019, before increasing again to 70.53 in 2021 (online supplemental figure S14 and table S9). Similar patterns were observed across endemic groups and seasons, except in endemicity group 2 (MIR declined over 2017–2020) and in endemicity group 3 and during the dry season (MIR decreased slightly over 2019–2020) (online supplemental figure S14). MIR in endemicity group 1 were over 10 times higher than in endemicity groups 2 and 3 (online supplemental figure S14). District-level temporal trends generally followed those of their corresponding endemicity group, with a few exceptions (figure 3b). For instance, while several districts followed the overall increase in MIR between 2017 and 2018 (eg, districts in Tambacounda, Kolda, Matam, Sédhiou, Kaffrine), others showed a decrease (eg, districts in Fatick, Thiès, Ziguinchor) (online supplemental table S10). Seasonal MIR estimates are shown in online supplemental figure S15.

### Model performance validation

Bayesian geostatistical models of MIR implemented with forward stepwise variable selection were validated using cross-validation schemes. In random cross-validation, BM performed well (r²=0.65), with WM performing slightly better (r²=0.67) and DM showing lower performance (r²=0.56) (figure 3a and online supplemental table S7). Among endemicity models, E2 showed the best performance (r²=0.69), followed by E1 (r²=0.43) and E3 (r²=0.32) (online supplemental table S7). Endemicity models achieved similar performance to BM applied within endemicity groups (ie, using the same model, training and test sets), denoted as BM for E1–E3 (figure 3a). BM for E1 outperformed E1, while E2 and E3 outperformed BM (figure 3a). Other cross-validation metrics (MAE and R²) are shown in online supplemental tables S7 and S8 and online supplemental figure S10. Cross-validation results of intermediate model outputs (eg, DHS indicators) are reported in online supplemental tables S5 and S6.

### Posterior parameter estimates

Posterior estimates of the MIR model parameters are presented in table 1. Associations were considered significant when the 95% credible interval of the regression coefficients excluded zero.

**Table 1:** Posterior estimates of Bayesian geostatistical MIR models

Variable	Mean (95% credible interval)					
	BM	WM	DM	E1	E2	E3
Intercept ($\beta_{0}$)	−3.61 (−4.40 to −2.82)	−4.00 (−4.72 to −3.28)	−4.37 (−5.12 to −3.62)	−1.24 (−2.20 to −0.32)	−5.98 (−7.24 to −4.72)	−4.56 (−5.44 to −3.67)
Precipitation	–	–	–	0.45 (−0.06 to 0.96)	1.64 (0.95 to 2.34)	0.17 (−0.05 to 0.38)
Daytime LST	−0.37 (−0.50 to −0.23)	−0.34 (−0.48 to −0.19)	–	−1.60 (−2.08 to −1.12)	–	−0.14 (−0.26 to −0.02)
Night-time LST	0.26 (0.15 to 0.37)	0.24 (0.13 to 0.36)	–	–	–	–
NDMI	–	–	–	−0.50 (−0.70 to −0.30)	0.17 (−0.08 to 0.42)	–
Prop. of bare land	–	0.003 (−0.08 to 0.07)	–	–	0.22 (0.09 to 0.35)	−0.08 (−0.15 to −0.02)
Prop. of built-up	0.57 (0.52 to 0.62)	0.51 (0.44 to 0.58)	0.63 (0.58 to 0.67)	0.43 (0.28 to 0.58)	0.63 (0.52 to 0.75)	0.53 (0.46 to 0.60)
Prop. of cropland	0.02 (−0.03 to 0.07)	–	0.04 (−0.01 to 0.10)	–	0.35 (0.20 to 0.50)	–
Prop. of flooded vegetation	–	−0.10 (−0.16 to −0.05)	–	1.15 (−0.22 to 2.53)	–	–
Prop. of shrubland	–	−0.11 (−0.19 to −0.02)	–	−0.38 (−0.50 to −0.26)	–	–
Prop. of trees	0.11 (0.05 to 0.17)	–	0.15 (0.09 to 0.22)	–	–	–
Prop. of water	–	–	–	−1.23 (−1.76 to −0.69)	–	–
Elevation	–	–	−0.13 (−0.23 to −0.03)	–	−0.58 (−0.95 to −0.21)	–
Access to basic sanitation	0.23 (0.16 to 0.30)	0.25 (0.18 to 0.32)	0.18 (0.11 to 0.25)	–	–	0.18 (0.09 to 0.27)
Prop. of Fula	–	–	–	–	−0.31 (−0.59 to −0.04)	–
Stunting in children	0.26 (0.17 to 0.35)	0.29 (0.20 to 0.37)	0.25 (0.16 to 0.33)	0.40 (0.26 to 0.54)	0.32 (0.09 to 0.54)	0.25 (0.14 to 0.37)
Distance to major roads	−0.19 (−0.25 to −0.13)	−0.12 (−0.18 to −0.06)	−0.13 (−0.19 to −0.07)	–	–	−0.21 (−0.28 to −0.14)
Walking time to health facilities	−0.35 (−0.41 to −0.28)	−0.34 (−0.41 to −0.28)	−0.38 (−0.45 to −0.31)	−0.23 (−0.30 to −0.16)	−1.84 (−2.17 to −1.51)	−0.36 (−0.45 to −0.27)
Precision of uncorrelated random variations ($\sigma_{e}^{-2})$)	1.12 (1.08 to 1.17)	1.17 (1.13 to 1.22)	1.09 (1.05 to 1.13)	2.27 (2.05 to 2.50)	1.02 (0.95 to 1.10)	1.15 (1.09 to 1.20)
Range (r)	2.16* (1.71 to 2.73)	2.22* (1.74 to 2.76)	2.01* (1.59 to 2.53)	1.74* (1.13, 2.57)	0.91* (0.59 to 1.40)	2.17* (1.59 to 2.87)
SD of spatially correlated variations ($\sigma_{w})$)	2.78 (2.22 to 3.46)	2.53 (2.00 to 3.10)	2.57 (2.06 to 3.19)	1.58 (1.10 to 2.21)	1.80 (1.31 to 2.46)	2.46 (1.83 to 3.21)
Autoregressive coefficient (a)	0.99 (0.98 to 0.99)	0.98 (0.97 to 0.99)	0.99 (0.98 to 0.99)	0.97 (0.95 to 0.99)	0.97 (0.94 to 0.99)	0.98 (0.96 to 0.99)

* These correspond to ranges of approximately 240, 247, 223, 193, 101 and 241 km respectively (1° = 111.12 km). Posterior estimates are reported as the mean and the 95% credible interval (2.5% to 97.5%). Note that the interpretation of the SD of structured and unstructured log-scale effects depends on incidence magnitudes; small absolute changes at low incidence can imply large multiplicative shifts on the log scale.

BM, benchmark model; DM, dry season model; LST, land surface temperature; MIR, malaria incidence rate; NDMI, normalised difference moisture index; Prop., proportion; SD, standard deviation; WM, wet season model.

Three covariates were consistently selected across all models: the proportion of built-up areas (positive association), child stunting prevalence (positive association) and walking time to health facilities (negative association). Most covariates selected in WM and/or DM also appeared in BM (table 1), but some were season-specific. For example, MIR significantly decreased with the proportion of shrubland and flooded vegetation only in WM, while higher elevation was associated with lower MIR only in DM. MIR showed a significant negative association with daytime land surface temperature (LST) and a positive association with night-time LST in both BM and WM (table 1).

Greater variability in covariate selection was observed in the endemicity models, and they included covariates that were not selected at the national level. In E1, MIR was negatively associated with daytime LST, normalised difference moisture index (NDMI), proportion of water and shrubland (table 1). In E2, significant positive associations were found with precipitation, proportion of cropland and bare land, while negative associations were observed with elevation and proportion of Fula. In E3, covariates were largely similar to those in the national-level models (table 1). Some covariates showed opposite relationships with MIR across endemicity levels (eg, NDMI and the proportion of bare land).

### Dasymetric disaggregation

**Figure 4:**
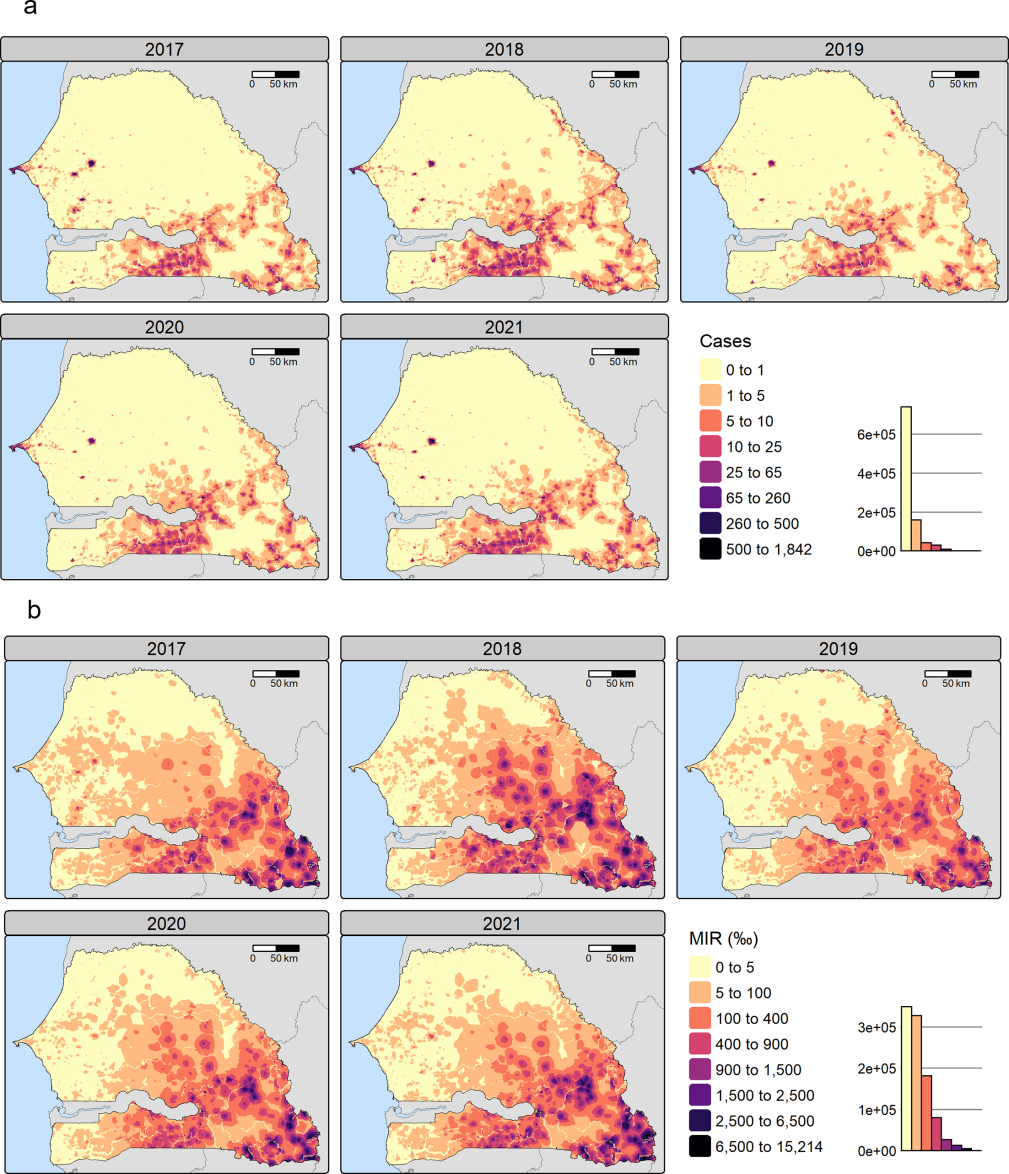
Disaggregated (**a**) reported cases and (**b**) reported malaria incidence rates (MIR, per 1000 people) for 2017–2021 at 1 km resolution. In (**a**), cases per pixel were obtained by dasymetric disaggregation of health facility cases. In (**b**), reported MIR were calculated by dividing disaggregated cases by the pixel-level treatment-seeking population. Estimates use BM and WorldPop’s unconstrained population counts. MIR above 1000‰ reflect malaria repeat episodes^53^ and migration.^54^ BM, benchmark model; MIR, malaria incidence rate.

Dasymetric disaggregation was used to redistribute HF cases to 1 km pixels (figure 4). Hotspots in reported malaria cases were found in densely populated areas (Dakar, Diourbel and Kaolack) and in the KKT area (figure 4a and online supplemental figures S19 and S21). When adjusted for population, Dakar, Diourbel and Kaolack showed lower MIR compared with the KKT area, where it sometimes exceeded 1000‰ (eg, along national borders). Reported MIR generally decreased concentrically around health facilities (figure 4b), with similar trends in both wet and dry seasons (online supplemental figures S20a and S22b). However, performing the disaggregation using constrained population counts eliminated these patterns (online supplemental figure S18, S20b and S22b). In Dakar, the highest number of cases was observed in the western side (Dakar-Centre) as well as in Pikine, Keur Massar and Rufisque (online supplemental figure S16; city locations in online supplemental figure S27). After population adjustment, MIR were relatively higher in Pikine, Keur Massar and Rufisque, and in the eastern side (online supplemental figure S17).

As a consistency check, regional reported cases matched the sum of disaggregated cases per region (online supplemental figure S24). The comparison with DHS prevalence estimates provided an external plausibility check of the incidence maps. While prevalence and reported clinical incidence measure different epidemiological quantities (influenced by factors such as immunity and healthcare access and reflecting different age groups), incidence increased with child prevalence up to approximately 10%, after which interpretation became uncertain due to the limited number of survey cluster data points (online supplemental figure S25).

## Discussion

We modelled malaria incidence in Senegal (2017–2021) using routine DHIS2 data and high-resolution environmental and socioeconomic covariates within a Bayesian hierarchical modelling framework. Separate models were fitted by season and endemicity to account for variation due to transmission level. We also used dasymetric disaggregation^34 35^ to redistribute reported malaria cases from health facilities to a 1 km spatial resolution.

### Risk factor profiles by transmission level

Across all endemic groups and seasons, reported malaria incidence was associated with urban-related covariates: it increased near built-up areas (in line with previous work^61 62^), and decreased away from health facilities (as previously reported^5 63^) and roads. These patterns reflect higher reported incidence in urban areas due to missing geolocation of rural health facilities, higher treatment-seeking in cities^41^ and a larger burden of imported cases.^5^ While seasonal variation was limited (in line with previous work^64^), some climate-related covariates were only relevant during the rainy season, when rainfall and temperature peaks coincide. In the wet season, incidence declined with higher daytime LST—likely exceeding mosquito thermal limits^65^—while higher night-time LST was associated with increased incidence, suggesting higher mosquito survival in areas retaining heat at night (eg, water^66^). Flooded vegetation showed a negative association, possibly because wetlands are more prevalent in low-transmission areas.

Risk factors varied more strongly across endemicity groups, also reflecting differences in urbanisation. Several risk factors were only relevant in specific settings. Daytime LST showed negative associations in high and low-transmission areas but was less relevant in cooler western urban areas.^67^ Flooded vegetation and shrubland emerged only in high-transmission rural areas (the KKT area), while the Fula ethnic group and cropland were only relevant in western urban areas (eg, Dakar). These associations align with previous work: breeding sites typically develop in rainfed croplands within Senegalese cities,^68 69^ whereas the Fula ethnic group (negatively associated) is genetically less susceptible to malaria.^70 71^ Some associations reversed by context. In the KKT area, incidence decreased with NDMI and water, reflecting low transmission in Niokolo-Koba National Park (figure 1a), through which most rivers flow. In contrast, in highly urbanised areas (eg, Dakar), incidence increased with rainfall and NDMI, likely due to breeding sites in flooded construction sites.^68 69 72–74^

Differences between national and endemicity models further highlight risk factor heterogeneity. Some covariates only emerged in endemic-specific models, likely because strong local relationships are diluted at national level. Conversely, national models also selected covariates that are not relevant everywhere and may be biased towards low-transmission areas, which are overrepresented in the dataset (see online supplemental table S4). This likely explains the similarity between national model covariates and those in the model for low-transmission areas. Also in Senegal, previous work has found that national population models underrepresented rural areas^75^ and that malaria drivers differed according to scale.^44^ Cross-validation results also support this. Endemic-specific models often outperformed the national model within their respective areas (figure 3a and online supplemental figure S10), suggesting that local models capture fine-scale structures that are smoothed over in the national model.

Overall, our findings support previous studies highlighting the heterogeneity of malaria drivers by endemicity level^76^ and geographical context.^8 9 16 18 77^ Understanding these context-specific drivers is essential for designing targeted, locally adapted interventions.^23^ Future studies could further examine how risk factors vary according to transmission level by incorporating season-specific or endemic-specific parameters within a single model.

### Spatiotemporal patterns of malaria incidence

Modelled malaria incidence increased from 60‰ to 76‰ between 2017 and 2018, decreased to 48‰ in 2019, and increased again to 71‰ in 2021. The 2018 rise may reflect the absence of mass ITN distribution, seasonal malaria chemoprevention (SMC) and indoor residual spraying (IRS) campaigns that year,^78–80^ while the 2019 drop corresponds to renewed intervention efforts^80^ (online supplemental table S11). The subsequent rise in 2020–2021 aligns with COVID-19-related disruptions^81 82^ and unusually heavy rainfall.^82^ Incidence trends from 2017 to 2021 broadly mirror ITN coverage patterns (online supplemental figure S26), though our models did not identify this relationship, potentially due to spatiotemporal confounding. Our results underscore the importance of continued malaria interventions to sustain progress and achieve elimination. Although we did not consider interventions other than ITNs, primarily due to a lack of fine-scale data, their effects are likely to be reflected in the temporal random effects. Future work could integrate covariates on IRS and artemisinin-based combination therapies (ACT) use from DHS data, provided that spatial interpolation challenges related to short survey reference periods (eg, ACT use in the 2 weeks prior to the survey) are addressed for the latter.^83^

Fine-scale maps of reported malaria incidence revealed a clear increasing gradient from north-west to south-east. In line with previous work,^44^ high-risk areas were located along national borders, with incidence exceeding 1000‰, partly due to internal migration^84^ and immigration in the Economic Community of West African States (ECOWAS).^54^ These incidence rates also reflect repeated infections, with 5%–10% of cases in children occurring within 42 days of a previous episode,^53^ a figure likely underestimated because data come from low-transmission regions.^53^ Urban hotspots were identified in Dakar, Diourbel and Kaolack, consistent with previous studies^9 44 68 69 73 85^ and the rising urban malaria burden in SSA.^6–9 86^ Reported malaria incidence decreased concentrically around health facilities and urban centres (figure 4b), suggesting potential underdiagnosis in remote areas—consistent with Senegal’s low treatment-seeking rates.^1 87^ However, this pattern may also reflect a visual artefact from disaggregating cases across all pixels, including unpopulated areas. In unconstrained population surfaces, uninhabited pixels are assigned population estimates. As these pixels are typically far from facilities, they are predicted to have very low posterior MIR based on the estimated associations. As a result, they receive few cases in the dasymetric disaggregation, leading to small but non-zero reported MIR estimates in uninhabited areas. Meanwhile, neighbouring inhabited pixels may have deflated population denominators, inflating MIR locally (eg, see the red spots in online supplemental figure S23c). Together, this creates concentric rings around facilities, which disappear when disaggregation is restricted to populated areas (online supplemental figure S23).

The comparison of our incidence estimates with malaria prevalence in children (0–5 years) at the DHS cluster level (online supplemental figure S25) revealed a similar relationship to that observed in previous work modelling the relationship between prevalence in children aged 2–10 years and incidence in older age groups.^88^ Our incidence estimates also largely reflect adolescents and adults, who account for most reported cases in Senegal.^89^ As expected, incidence and prevalence estimates did not match perfectly, as DHS prevalence and routine reported clinical incidence measure different aspects of malaria epidemiology. Senegal implements SMC annually, reducing prevalence in treated children,^24 25^ while adolescents and adults can experience higher clinical incidence.^24 25^ In low-transmission settings, survey prevalence is also underestimated due to excess zeros.^26 27^ Conversely, high prevalence in children may reflect remote, non-SMC areas, where adults are asymptomatic,^26 90^ resulting in low clinical incidence. Future studies will integrate routine data with prevalence surveys to leverage their complementary strengths.^11 31 61 91^

Our fine-scale malaria incidence rates were estimated using dasymetric disaggregation, a technique widely used in population mapping. Although we implemented this approach within a Bayesian framework, it can also be applied with simpler models—such as linear regression—which may be more intuitive and easier to operationalise in policy settings.^45^ In recent years, other disaggregation regression methods involving pixel-level inference at the modelling stage have been used to produce fine-scale estimates of malaria incidence.^3 5 33 42 61 92 93^ Future research should explore how these methods compare with dasymetric disaggregation. Future studies will also focus on disaggregating fine-scale incidence estimates by sex/age groups to enable more robust age-specific interventions.

### Limitations

Although our model accounted for several routine-data biases (eg, access to healthcare, under-reporting), other biases remain, especially in rural areas such as RDT stockouts and the higher prevalence of asymptomatic infections.^26^ These factors can contribute to the spatial patterns in our maps and the relationships found with urban covariates (eg, negative association with travel time to health facilities). Even with adjustments, reported incidence estimates may still miss individuals who do not seek care. For this reason, the incidence maps should be interpreted alongside indicators of healthcare access. Online supplemental figure S28 shows how combining reported incidence with healthcare accessibility can distinguish areas where low reported incidence is more likely to reflect underdiagnosis, poor access to care or unmet need than a genuinely low malaria burden. Such areas could be prioritised for enhanced surveillance (eg, identification of asymptomatic reservoirs) or community-level interventions.

Cases were reported to both the DHIS2 and the Excel-based system of the National Malaria Control Programme during the study period,^38^ potentially leading to missing cases, although the DHIS2 has increasingly replaced the Excel-based system since 2016.^38^ Missing cases were treated as unreported, although they may indicate no transmission^38^ in low-transmission settings and in the dry season,^38^ likely contributing to lower model performance (figure 3a). We were also unable to distinguish local from imported cases.^26^ These biases likely explain the relatively high standard deviation of the unstructured random effects (table 1), particularly in low-transmission settings. When incidence is low (0–1 reported case), a single unreported or imported case can substantially inflate residual variability. Tiny absolute changes in very low, highly right-skewed incidence values translate into large relative shifts on the log-incidence scale, making the variability appear higher.

Catchment areas were based on walking accessibility, although some populations use other transport modes.^47^ Relative rather than absolute travel times are less likely to be affected by transport mode,^32^ but future work could investigate multimodal approaches. We also assumed seasonally invariant catchment populations, although travel time,^24^ population denominators^94^ and treatment-seeking behaviour^24^ vary seasonally. In addition, when malaria persists in residual hotspots in low-transmission settings^2 36 95 96^ or in the dry season,^44^ catchment-level averaging may smooth out local conditions. Finally, our results may be subject to ecological fallacy,^97^ since the models are trained at catchment level but used to infer pixel-level patterns. This is partly mitigated by ensuring that pixel-level cases sum to catchment-level case counts during the dasymetric disaggregation process, rather than relying solely on model-based predictions.^34^

## Conclusion

We analysed spatiotemporal trends in malaria incidence in Senegal (2017–2021) using routine DHIS2 data and high-resolution covariates within a Bayesian hierarchical modelling framework. To capture heterogeneity in risk factors, we fitted separate models by season and endemicity level. While seasonal variation was limited, key risk factors were uniquely associated with low (eg, distance to roads), moderate (eg, cropland) and high-transmission settings (eg, flooded vegetation). Using dasymetric disaggregation, we redistributed reported facility cases to 1 km pixels, generating high-resolution maps of reported malaria incidence. Rather than representing the total malaria burden, these maps reflect malaria cases routinely diagnosed and reported through the public-sector health system and revealed potential underdiagnosis in remote areas. With prevalence surveys becoming scarcer following the closure of the United States Agency for International Development (USAID), and DHIS2 increasingly adopted across SSA, our approach offers a scalable alternative for malaria-endemic countries with routine case data. As transmission becomes increasingly heterogeneous, this method helps identify critical, endemic-specific risk factors and produce fine-scale incidence maps to drive more effective intervention strategies and, when interpreted alongside healthcare accessibility maps, identify areas of potential underdiagnosis.

## Supplementary material

10.1136/bmjgh-2025-023003 online supplemental file 1

10.1136/bmjgh-2025-023003 online supplemental file 2

10.1136/bmjgh-2025-023003 online supplemental file 3

## Data Availability

DHS data for Senegal can be accessed through the DHS Program upon registration and request at https://dhsprogram.com/data/dataset_admin/login_main.cfm.
