# Supplementary material for: Where and why malaria persists in heterogeneous transmission settings: insights from routine health facility data
Source: BMJ Glob Health. 2026 Aug 7;11(8):e023003. doi: 10.1136/bmjgh-2025-023003 (PMC13475453; doi:10.1136/bmjgh-2025-023003)
Supplement: Supplementary file 1 [file bmjgh-11-8-s001.docx]

Supplementary File 1: Additional details on the data and methods

# Routine malaria data

**Figure S1.** Distribution of public health facilities used in this study (n = 1472).

**Table S1.** Number of public health facilities, percentage geolocated and reporting rate by region

| **Region** | **Total number of facilities** | **Geolocated facilities** | **Percentage geolocated (%)** | **Reporting rate (%)** |
| --- | --- | --- | --- | --- |
| Dakar | 168 | 163 | 97.0 | 93.7 |
| Diourbel | 107 | 97 | 90.7 | 91.7 |
| Fatick | 128 | 114 | 89.1 | 98.8 |
| Kaffrine | 99 | 78 | 78.8 | 100.0 |
| Kaolack | 121 | 112 | 92.6 | 98.4 |
| Kédougou | 48 | 37 | 77.1 | 100.0 |
| Kolda | 82 | 64 | 78.1 | 100.0 |
| Louga | 128 | 122 | 95.3 | 100.0 |
| Matam | 107 | 105 | 98.1 | 99.5 |
| Saint-Louis | 132 | 124 | 93.9 | 100.0 |
| Sédhiou | 63 | 58 | 92.1 | 100.0 |
| Tambacounda | 151 | 116 | 76.8 | 99.7 |
| Thiès | 186 | 170 | 91.4 | 95.9 |
| Ziguinchor | 123 | 112 | 91.1 | 99.9 |

*Note*. Public health facilities considered here are those with at least one reported case of malaria over the period 2017–2021. Reporting rates are given for 2021.

**Figure S2.** Malaria reported cases per health facility over 2017-2021.

# Geospatial covariates

All covariates used in this study are listed in Table S2 and described in the following sections.

**Table S2.** Geospatial covariates and their characteristics

| **Category** | **Variables** | **Date** | **Spatial resolution** | **Source** |
| --- | --- | --- | --- | --- |
| Climate & weather | Precipitation | Climatic average over 1988-2018 | 1000 m | CHELSA [1] (<https://chelsa-climate.org/>) |
|  | Daytime land surface temperature (LST), night-time LST | Annual (2017-2021) | 1000 m | MODIS [2,3] (<http://modis.gsfc.nasa.gov/>) |
| Vector-habitat | Normalized difference moisture index (NDMI) | Annual (2017-2021) | 10 m | Computed from Sentinel-2 L1C composites of the Joint Research Centre (JRC) [4] (<https://forobs.jrc.ec.europa.eu/sentinel/sentinel2_composite>) |
|  | Land use and land cover: water, trees, flooded vegetation, cropland, grassland, bare land, shrubland, built-up | Annual (2017-2021) | 10 m | Dynamic World by Google and the World Resources Institute [5] (<https://dynamicworld.app/>) |
|  | Elevation | 2000 | 30 m | US Geological Survey [6] (<http://eros.usgs.gov/elevation-products>) |
| Susceptibility | Access to basic sanitation service, proportion of Fula, stunting in children | Annual (2017-19/20 avail.; stunting: 2020-21 proj., others: 2021 proj.) | Survey clusters | The DHS Program |
| Resilience | Distance to major roads | 2023 | 100 m | OpenStreetMap ([www.openstreetmap.org](http://www.openstreetmap.org)) |
|  | Walking-only travel time to health facilities | Annual (2017-2021) | 1000 m | Walking time is computed using elevation, land use, roads and rivers (see section Computation of travel time) |
|  | Ownership of insecticide-treated nets, literacy rate in women | Annual (2017-20 avail.; 2021 proj.) | Survey clusters | The DHS Program |

*Note.* Avail. and proj. stand for available and projected, respectively.

## Remotely sensed covariates

### Precipitation

We calculated the mean year-monthly precipitation over period 1988-2018 from CHELSA (Climatologies at high resolution for the earth’s land surface areas) v2.1 climate data [1]. CHELSA are statistically downscaled model output estimates at 30 arcsec (~1 km). While showing similar accuracy to WorldClim temperature estimates, previous work shows that CHELSA precipitation data are more accurate [1] and should be preferred to interpolated data (i.e. WorldClim) in areas where weather stations are sparsely distributed [1,7]. For replicability of this work beyond Senegal, we used CHELSA climate data over other existing products.

### Land surface temperature

From MODIS Aqua and Terra data collections (MYD11A1 v006 and MOD11A1 v006) [2,3], we extracted the mean monthly daytime and night-time land surface temperature (LST) at 1 km resolution for the years 2017 to 2021.

### Normalized difference moisture index

The normalized difference moisture index (NDMI) was computed from the 2017 to 2021 Sentinel-2 L1C cloud-free composites (10 m resolution) of the Joint Research Centre (JRC) [4]. The methodology to create these Sentinel-2 L1C composites is tailored to the pan-tropical zone (e.g. Senegal) where the large cloud cover sometimes leads to the lack of cloud-free images for months [4]. Although not atmospherically corrected, these composites can be used directly for extracting vegetation indices, as done in [8], as these are less influenced by atmospheric effects [4,8]. So far, existing Sentinel-2 L2A composites (e.g. the Sentinel-2 Global Mosaic service) are often prone to errors due to the quality of the scene classification of Sentinel-2 L2A data and cloud masks provided by the Copernicus service [4].

### Land use land cover

Land use land cover (LULC) data were extracted from the Dynamic World global LULC dataset available in near-real time at 10 m spatial resolution. This dataset is derived from Sentinel-2 satellite imagery using deep learning algorithms. It provides the classification probability of nine LULC categories: water, trees, flooded vegetation, crops, grass, built area, bare land, shrubland, and snow [5]. In this study, the snow category was discarded. All LULC data were yearly (2017-2021) aggregated using the most frequent LULC class on average. Classification validation shows an overall agreement of 73.8 % with experts, outperforming current global LULC products such as ESA CCI 2018 and ESA CGLS ProbaV 2019 [5].

### Distance to major roads

Distance (100 m resolution) to major roads (i.e. motorways, trunks, primary, secondary, and tertiary roads) were computed using the most recent OpenStreetMap data available at the time of the study.

### Elevation

A Digital Elevation Model at 30 m spatial resolution was obtained from the NASA Shuttle Radar Topography Mission (SRTM) [6].

## DHS indicators

In this study, we used data from the most recent household surveys conducted in Senegal: the 2017, 2018, 2019 continuous DHS, the 2020-2021 Malaria Indicator Survey (MIS), and the 2023 continuous DHS. We selected several indicators that influence vulnerability to malaria: proportion of Fula people, access to basic sanitation, stunting in children, insecticide-treated net (ITN) ownership and literacy rate in women. These indicators were aggregated at the cluster level following the instructions of the survey reports and the DHS program (40). They are listed in Table S2 and described in the following sections.

### Proportion of Fula

This indicator shows the proportion of the population (men and women) belonging to the Fula ethnic group. Fula, Fulani or Pulaar have been shown to be less susceptible to malaria due to higher antibody levels and other protective effects (type O blood, etc.) [9]. In addition, the nomadic lifestyle of some Fula ethnic groups in Senegal may also influence malaria prevention behaviour.

### Stunting in children

Stunting is a measure of chronic (i.e. repeated) nutritional deficiency. For children, it is measured based on the children height for a specific age and sex. This indicator is defined as the proportion of children under five who are moderately or severely stunted, i.e. children whose height-for-age is less than two standard deviations from the median defined by the Child Growth Standards of the World Health Organisation (WHO) [10–12].

### Access to basic sanitation service

This indicator is defined as the proportion of households having access to a basic sanitation service. According to the WHO/UNICEF Joint Monitoring Programme classification standards for Water Supply and Sanitation, basic sanitation services are characterised as the use of improved sanitation facilities that are not shared with any other households [13]. Note that improved sanitation facilities are toilet facilities that hygienically separate excreta from human contact (i.e. flush to piped sewer system/septic tank/pit latrine, ventilated improved pit latrine, pit latrine with slab and composting toilet) [10,11].

### Literacy rate in women

The literacy rate in women is the proportion of women aged 15 to 49 who are literate. In DHS, literacy is assessed by the interviewer by asking the respondent to read a sentence or part of a sentence in English or in the respondent native language. In this study, we defined literate women as those with more than secondary school education or women who can read at least part of a sentence, following [11,12].

### Insecticide-treated net coverage

Insecticide-treated nets (ITN) include all types of mosquito nets, including those that need to be treated/retreated and those that are factory treated with an efficacy of about three years (known as long-lasting insecticidal nets (LLIN)), which are the most recommended [14]. Here we use ITN to refer to LLIN only. In this study, we used the proportion of households with at least one ITN.

### Creation of interpolated surfaces

To generate interpolated surfaces for these DHS indicators, we used spatio-temporal Bayesian models without covariates to avoid circularity issues when later modelling malaria incidence, as recommended by [15]. Consider $Y_{it}$ as the number of individuals with a given condition or characteristic of interest (e.g. stunted children, households with at least one ITN, etc.) out of $N_{it}$, the number of individuals surveyed in survey cluster $i$ $\left( i=1,\ldots,n \right)$ for year $t$ $(t=1,\ldots, 7$, corresponding to the years 2017 to 2023). Given the true proportion $p_{it}$, $Y_{it}$ follows a binomial distribution:

$$\left. Y_{it} \right|p_{it}\sim Binomial\left( N_{it}, p_{it} \right)$$

$$\text{logit}\left( p_{it} \right)=\beta_{0}+w_{i}+\phi_{t}$$

(S1)

where $\beta_{0}$ is the intercept. $w_{i}$ is a spatially structured random effect modelled as a zero-mean Gaussian Process (GP) such that $w_{i} \sim GP\left( 0, \Sigma\right)$ [16]. Spatial dependence between two observations of the GP at locations $s_{1}$ and $s_{2}$can be modelled using the Matérn covariance function $\Sigma$, defined as follows [16]:

$$C\left( s_{1},s_{2} \right)= \frac{2^{1-\nu}\sigma_{w}^{2}}{\Gamma\left( \nu\right)}\left( \kappa h \right)^{\nu}K_{v}(\kappa h)$$

(S2)

where $h$ is the Euclidean distance between $s_{1}$ and $s_{2}$, $\sigma_{w}^{2}$ is the marginal variance, $\nu>0$ controls the smoothness of the spatial process, $K_{v}$ is the modified Bessel function of the second kind and $\kappa>0$ is a scale parameter controlling the range, i.e. the distance from which there is no more spatial autocorrelation in the data. The range $r$ is given as a function of $\kappa$ such that $r=\sqrt{8\nu}/\kappa$. In this paper, we set $\nu=1$.

$\phi_{t}$ is the temporally structured random effect, modelled as an autoregressive process of order 1 (AR(1)) with $\phi_{t}\sim a\phi_{t-1}+\epsilon_{t}$ and $\phi_{1}\sim N\left( 0, \frac{\sigma_{\epsilon}^{2}}{1-a^{2}} \right)$ [16,17]. $\left| a \right|<1$ is the autoregressive coefficient and $\epsilon_{t}$ is a zero-mean Gaussian noise term with variance $\sigma_{\epsilon}^{2}$. Weekly informative priors were assigned to parameters and hyperparameters: $\beta_{0}\sim N(0, \infty)$, $P\left( r<d_{max} \right)=0.95$, $P\left( \sigma_{w}>5 \right)=0.05$, $log(\frac{1+a}{1-a})\sim N(0, {0.15}^{-1})$ and $\log\left( \sigma_{\epsilon}^{-2}\left( 1-a^{2} \right) \right)\sim\mathrm{lo}\text{g-ga}\mathrm{mma}\left( 1, 5\times{10}^{-5} \right)$. $d_{max}$ is the maximum distance (in degrees) measured across the study area.

The 2020–2021 MIS data were primarily treated as representing 2020, and interpolated surfaces were projected for 2021 using the model. Model performance was evaluated using two cross-validation techniques: 1) 5-repeated 5-fold random cross-validation and 2) temporal block cross-validation (i.e. leaving out one year of observations at a time to assess the model's ability to predict over time) following [18]. The following metrics were computed on the posterior mean estimates of the test sets: the mean absolute error (MAE), the root mean square error (RMSE), the coefficient of determination (R²) and the squared correlation between predicted and observed values (r²).

# Catchment population

## Computation of travel time

Following [19], travel speed $v$ (km/h) was calculated as a function of slope (derived from a digital elevation model [6]) using Tobler’s hiking function [20], assuming an individual walks at 5 km/h on a flat surface:

$$v=6e^{-3.5\left| \tan\left( \frac{\text{slope in degrees}}{57.296} \right)+0.05 \right|}$$

(S3)

Travel speeds were then adjusted based on land cover type following [21] (see Table S3). Lastly, using the *gdistance* package in R statistical programming software, travel speeds were converted into conductance values (i.e. the inverse of travel time), which were then used to calculate gridded travel time estimates to health facilities (from 2017 to 2021) using a least cost algorithm (*accost* function).

**Table S3.** Land and road type and associated walking speed in km/h

| **Data source** | **Land and road type** | **Walking speed (km/h)** |
| --- | --- | --- |
| Dynamic World LULC | Built up | 5 |
|  | Trees | 3.5 |
|  | Shrubland | 4.5 |
|  | Cropland | 3.5 |
|  | Grassland | 4 |
|  | Bare ground | 5 |
|  | Flooded vegetation | 0 |
|  | Water bodies | 0 |
| OpenStreetMap | Rivers | 0 |
|  | Roads | 5 |

*Note.* Walking speeds are based on [21]. LULC stands for land use land cover.

## Treatment-seeking probability

To account for treatment-seeking behaviour in estimating catchment populations, we used data on attendance at public health facilities among children with fever from the 2017, 2018, 2019 and 2023 DHS and the 2020-2021 MIS. The random variable $z_{it}$ follows a Bernoulli distribution with probability $p_{it}$ of attending a public health facility for a child with fever $i$ $\left( i=1,\ldots,n \right)$ in year $t (t=1,\ldots, 7$, corresponding to the years 2017 to 2023). The model is expressed as follows:

$$\text{logit}\left( p_{it} \right)=\beta_{0}+d_{it}\beta+w_{i}+\phi_{t}$$

(S4)

where $\beta_{0}$ is the intercept, $d_{it}$ is the minimum walking time to a public health facility from location of child $i$ in year $t$ and $\beta$ is its regression coefficient, with $\beta\sim N(0, 1000)$ as prior. The rest of the parameters are defined as for Equation S1.

Model performance was assessed through 5-repeated 5-fold cross-validation and temporal block cross-validation by calculating the accuracy (proportion of correct predictions to all predictions), precision (proportion of true positives to all positive predictions), recall (proportion of true positives to all actual positives) and the Area Under Curve (AUC).

# Incidence models

Prior specification: Weekly informative priors were assigned to parameters and hyperparameters: $\beta_{0}\sim N(0, \infty)$, $\beta\sim N(0, 1000)$, $P\left( r<d_{max} \right)=0.95$, $P\left( \sigma_{w}>1 \right)=0.05$, $log(\frac{1+a}{1-a})\sim N(0, {0.15}^{-1})$ and $\sigma_{e}^{-2}\sim\text{Gamma}\left( 1, 5\times{10}^{-4} \right)$. $d_{max}$ is the maximum distance (in degrees) measured across the study area.

**Table S4.** Model names and their descriptions

| **Model name** | **Description** | **Number of facilities** |
| --- | --- | --- |
| Benchmark model (BM) | - $c_{jt}$: total number of cases over the year $t$ - Target: all health facilities | 1472 (1389) |
| Wet season model (WM) | - $c_{jt}$: total number of cases over the wet season of year $t$ (i.e. from June to Octobre) - Target: all health facilities | 1472 (1389) |
| Dry season model (DM) | - $c_{jt}$: total number of cases over the dry season of year $t$ (i.e. from Novembre to May) - Target: all health facilities | 1472 (1389) |
| Endemicity 1 model (E1) | - $c_{jt}$: total number of cases over the year $t$ - Target: health facilities from endemicity group 1 | 217 (216) |
| Endemicity 2 model (E2) | - $c_{jt}$: total number of cases over the year $t$ - Target: health facilities from endemicity group 2 | 373 (328) |
| Endemicity 3 model (E3) | - $c_{jt}$: total number of cases over the year $t$ - Target: health facilities from endemicity group 3 | 882 (845) |

*Note.* $c_{jt}$ is the number of malaria cases reported at facility $j$ in year $t$. The number of health facilities in brackets reflects the number after merging cases of facilities within the same 1×1 km grid cell during catchment area extraction.

# References

[1]	Karger DN, Conrad O, Böhner J, et al. Climatologies at high resolution for the earth’s land surface areas. Sci Data. 2017;4(1):170122.

[2]	Wan Z, Hook S, Hulley, G. MODIS/Terra Land Surface Temperature/Emissivity Daily L3 Global 1km SIN Grid V061 [dataset] [Internet]. NASA EOSDIS Land Processes DAAC; 2021 [cited 2023 May 10]. Available from: https://doi.org/10.5067/MODIS/MOD11A1.061.

[3]	Wan Z, Hook S, Hulley, G. MODIS/Aqua Land Surface Temperature/Emissivity Daily L3 Global 1km SIN Grid V061 [dataset] [Internet]. NASA EOSDIS Land Processes DAAC; 2021 [cited 2023 May 10]. Available from: https://doi.org/10.5067/MODIS/MYD11A1.061.

[4]	Simonetti D, Pimple U, Langner A, et al. Pan-tropical Sentinel-2 cloud-free annual composite datasets. Data in Brief. 2021;39:107488.

[5]	Brown CF, Brumby SP, Guzder-Williams B, et al. Dynamic World, Near real-time global 10 m land use land cover mapping. Sci Data. 2022;9(1):251.

[6]	Farr TG, Rosen PA, Caro E, et al. The Shuttle Radar Topography Mission. Reviews of Geophysics. 2007;45(2):RG2004.

[7]	Rusk J, Maharjan A, Tiwari P, et al. Multi-hazard susceptibility and exposure assessment of the Hindu Kush Himalaya. Science of The Total Environment. 2022;804:150039.

[8]	Verhegghen A, Kuzelova K, Syrris V, et al. Mapping Canopy Cover in African Dry Forests from the Combined Use of Sentinel-1 and Sentinel-2 Data: Application to Tanzania for the Year 2018. Remote Sensing. 2022;14(6):1522.

[9]	Seck MC, Thwing J, Fall FB, et al. Malaria prevalence, prevention and treatment seeking practices among nomadic pastoralists in northern Senegal. Malar J. 2017;16(1):413.

[10]	Gething P, Tatem A, Bird T, et al. Creating Spatial Interpolation Surfaces with DHS Data [Internet]. Rockville, Maryland, USA: ICF International; 2015. Available from: https://dhsprogram.com/pubs/pdf/SAR11/SAR11.pdf.

[11]	Croft TN, Marshall AMJ, Allen CK. Guide to DHS Statistics - DHS-7 [Internet]. Rockville, Maryland, USA: ICF; 2018. Available from: https://www.dhsprogram.com/pubs/pdf/DHSG1/Guide_to_DHS_Statistics_DHS-7_v2.pdf.

[12]	Bosco C, Alegana V, Bird T, et al. Exploring the high-resolution mapping of gender-disaggregated development indicators. J R Soc Interface. 2017;14(129):20160825.

[13]	World Health Organization, United Nations Children’s Fund (UNICEF). Progress on drinking water, sanitation and hygiene: 2017 update and SDG baselines [Internet]. Geneva: WHO and UNICEF; 2017 [cited 2023 May 15]. Available from: https://www.who.int/publications/i/item/9789241512893.

[14]	World Health Organization. WHO guidelines for malaria, 30 November 2024 [Internet]. Geneva: World Health Organization; 2024. Available from: https://doi.org/10.2471/B09146.

[15]	Morlighem C, Nnanatu CC, Visée C, et al. Spatial interpolation of health and demographic variables: Predicting malaria indicators with and without covariates. PLOS ONE. 2025;20(5):e0322819.

[16]	Rue H, Martino S, Chopin N. Approximate Bayesian inference for latent Gaussian models by using integrated nested Laplace approximations. Journal of the Royal Statistical Society: Series B (Statistical Methodology). 2009;71(2):319–392.

[17]	Gomez-Rubio V. Bayesian inference with INLA. New York: Chapman and Hall/CRC; 2020.

[18]	Epstein A, Namuganga JF, Nabende I, et al. Mapping malaria incidence using routine health facility surveillance data in Uganda. BMJ Global Health. 2023;8(5):e011137.

[19]	Alegana VA, Wright JA, Pentrina U, et al. Spatial modelling of healthcare utilisation for treatment of fever in Namibia. International Journal of Health Geographics. 2012;11(1):6.

[20]	Tobler W. Three Presentations On Geographical Analysis And Modeling: Non-isotropic Geographic Modeling; Speculations on the Geometry of Geography; and Global Spatial Analysis. Technical Report 93-1 [Internet]. National Center for Geographic Information and Analysis; 1993 [cited 2025 Feb 7]. Available from: https://www.researchgate.net/publication/2460331_Three_Presentations_On_Geographical_Analysis_And_Modeling.

[21]	Macharia PM, Ray N, Gitonga CW, et al. Combining school-catchment area models with geostatistical models for analysing school survey data from low-resource settings: Inferential benefits and limitations. Spatial Statistics. 2022;51:100679.
