# Supplementary material for: Where and why malaria persists in heterogeneous transmission settings: insights from routine health facility data
Source: BMJ Glob Health. 2026 Aug 7;11(8):e023003. doi: 10.1136/bmjgh-2025-023003 (PMC13475453; doi:10.1136/bmjgh-2025-023003)
Supplement: Supplementary file 2 [file bmjgh-11-8-s002.docx]

Supplementary File 2: Additional results

# Supplementary figures

**Figure S3.** Predicted access to basic sanitation service at 1 km spatial resolution for Senegal.

**Figure S4.** Predicted proportion of Fula people at 1 km spatial resolution for Senegal.

**Figure S5.** Predicted stunting prevalence in children under five at 1 km spatial resolution for Senegal.

**Figure S6.** Predicted ownership of insecticide-treated nets (ITN) at 1 km spatial resolution for Senegal.

**Figure S7.** Predicted literacy rate in women aged 15 to 49 at 1 km spatial resolution for Senegal.

**Figure S8.** Predicted access to healthcare at 1 km spatial resolution for Senegal.

**Figure S9.** Density plots of malaria incidence rates (MIR, per person-year) and their logarithmic transformation (log MIR).

**Figure S10.** Boxplot of performance metrics obtained using a random 5-repeated 5-fold cross-validation of Bayesian malaria incidence models. Mean absolute error (MAE) and R² values were calculated on the test sets (MIR scale). The R² is the coefficient of determination (COD). The middle horizontal line shows the median and dots indicate outliers.

**Figure S11.** Observed malaria incidence rates (MIR, per 1,000 people) per health facility for 2017–2021. MIR were calculated using all annually reported cases. Missing values correspond to health facilities that did not report cases in that year but had reported cases in any prior year. MIR values above 1000‰ likely reflect malaria repeat episodes [1] and migration [2].

**Figure S12.** Boxplot of malaria incidence rates (MIR, per 1,000 people) per health facility over 2017–2021. The middle horizontal line represents the median, while the dot represents the mean. Outliers are not shown to improve readability. The mean MIR is systematically higher than the median in the boxplot, reflecting a right-skewed distribution (Figure S9). Note that incidence rates above 1000‰ can be attributed to malaria repeat episodes [1] and migration [2]. Abbreviations: KKT (Kolda, Kédougou, Tambacounda), E1 (endemicity 1), E2 (endemicity 2), E3 (endemicity 3).

**Figure S13.** Posterior estimates of malaria incidence rates (MIR, per 1,000 people) per health facility for 2017–2021. These estimates are based on the benchmark model (BM).

**Figure S14.** Estimated malaria incidence rates (MIR, per 1,000 people) in Senegal from 2017–2021 by season and endemic group. Cases per health facility were first estimated by multiplying the observed MIR per health facility by the total potential catchment population (i.e. assuming 100% of the population sought health care for fever). For facilities with missing MIR data, we instead used the exponentiated Bayesian posterior mean log MIR from the relevant model (in order: BM, WM, DM, E1, E2, E3) to derive their estimated case counts. Cases were then summed and divided by the total population to calculate the annual MIR. Note that the y-axis in the endemicity group 1 panel differs from the other models.

**Figure S15.** Estimated malaria incidence rates (MIR, per 1,000 people) for 2017–2021 per health district. Cases per health facility were first estimated by multiplying the observed MIR per health facility by the total potential catchment population (i.e. assuming 100% of the population sought health care for fever). For facilities with missing MIR data, we instead used the exponentiated Bayesian posterior mean log MIR from the wet season (WM) and dry season (DM) models to derive their estimated case counts. Cases were then summed per health district and divided by the total district population to calculate the annual MIR. MIR above 1000‰ reflect malaria repeat episodes [1] and migration [2].

**Figure S16.** Disaggregated reported malaria cases for 2017–2021 at 1x1 km resolution in Dakar. Cases per pixel were obtained by a dasymetric disaggregation of the reported cases per health facility. These estimates are based on the benchmark model and WorldPop’s unconstrained population counts (i.e. population predicted across the entire country, including potential uninhabited areas).

**Figure S17.** Disaggregated malaria incidence rate (MIR, per 1,000 people) for 2017–2021 at 1x1 km resolution in Dakar. MIR were calculated by dividing disaggregated cases by the pixel treatment-seeking population. These estimates are based on the benchmark model and WorldPop’s unconstrained population counts (i.e. population predicted across the entire country, including potential uninhabited areas).

**Figure S18.** Disaggregated (a) reported cases and (b) malaria incidence rates (MIR, per 1,000 people) for 2017–2021 at 1×1 km resolution. In (a), cases per pixel were obtained by dasymetric disaggregation of cases reported by health facilities. In (b) MIR were calculated by dividing disaggregated cases by the pixel treatment-seeking population. Estimates are based on BM and WorldPop’s constrained population counts (i.e. population predicted only in settled areas). MIR above 1000‰ reflect malaria repeat episodes (53) and migration (54).

**Figure S19.** Disaggregated reported malaria cases for 2017–2021 at 1x1 km resolution in the wet season. (a) relies on unconstrained population counts from WorldPop (i.e. population predicted for the whole country, including potentially uninhabited areas), while (b) uses constrained population counts (i.e. population predicted only in settled areas). Cases per pixel were obtained by a dasymetric disaggregation of the reported cases per health facility. These estimates are based on the wet season model (WM).

**Figure S20.** Disaggregated malaria incidence rates (MIR, per 1,000 people) for 2017–2021 at 1x1 km resolution in the wet season. (a) relies on unconstrained population counts from WorldPop (i.e. population predicted for the whole country, including potentially uninhabited areas), while (b) uses constrained population counts (i.e. population predicted only in settled areas). Malaria incidence rates (MIR) were calculated by dividing disaggregated cases by the pixel treatment-seeking population. These estimates are based on the wet season model (WM).

**Figure S21.** Disaggregated reported malaria cases for 2017–2021 at 1x1 km resolution in the dry season. (a) relies on unconstrained population counts from WorldPop (i.e. population predicted for the whole country, including potentially uninhabited areas), while (b) uses constrained population counts (i.e. population predicted only in settled areas). Cases per pixel were obtained by a dasymetric disaggregation of the reported cases per health facility. These estimates are based on the dry season model (DM).

**Figure S22.** Disaggregated malaria incidence rates (MIR, per 1,000 people) for 2017–2021 at 1x1 km resolution in the dry season. (a) relies on unconstrained population counts from WorldPop (i.e. population predicted for the whole country, including potentially uninhabited areas), while (b) uses constrained population counts (i.e. population predicted only in settled areas). MIR were calculated by dividing disaggregated cases by the pixel treatment-seeking population. These estimates are based on the dry season model (DM).

**Figure S23**. Comparison of (a) unconstrained and (b) constrained malaria incidence rates (MIR, per 1,000 people) at 1×1 km resolution in Senegal in 2017. Fine‑scale incidence surfaces were produced by disaggregating catchment‑level case counts (a) across all pixels and (b) only across populated pixel (i.e. using WorldPop's unconstrained and constrained population counts, respectively). (c) displays the difference between unconstrained and constrained MIR estimates, with red areas indicating where unconstrained incidence is higher and blue areas indicating the opposite.

**Figure S24.** Comparison of reported and disaggregated malaria cases, aggregated by region (n = 14) over the period 2017–2021. Reported cases are aggregated from all health facilities within each region, while disaggregated cases are summed across all pixels within each region. The disaggregated cases per pixel were derived by disaggregating reported cases from health facilities. Missing reported cases were imputed using the respective models (in order: BM, WM, DM). The blue line represents the 1:1 line. As expected, all points lie close to this line, since dasymetric disaggregation ensures that the sum of pixel-level cases matches the total reported cases.

**Figure S25.** Relationship between predicted malaria incidence (cases per person-year) across all age groups and malaria prevalence in children under five in Senegal during the 2017 wet season. Cluster-level prevalence is based on the 2017 Senegal Demographic and Health Survey (DHS; mostly conducted during the rainy season), while fine-scale incidence estimates for the wet season (2017) were averaged within 5 km buffers around rural clusters and 2 km buffers around urban clusters. Each blue dot represents a DHS cluster (n = 400). The orange curve shows a quasi-Poisson generalized additive model (GAM) smoother (to account for overdispersion), which captures the non-linear association between prevalence and incidence. The green dashed line represents the linear regression fit (Pearson correlation coefficient = 0.328).

**Figure S26.** Percentage of households with at least one insecticide-treated net (ITN) in Senegal between 2017 and 2021, based on household survey estimates. Abbreviations: DHS (Demographic and Health Surveys), MIS (Malaria Indicator Surveys).

**Figure S27.** Major cities of Senegal.

For visibility purposes, the cities in the Thiès and Dakar regions are shown in panel (b). Cities are extracted from the World Cities Database [3], to which we added Keur Massar.

**Figure S28.** Bivariate map of malaria incidence rate (MIR) and healthcare access at 1 km resolution in Senegal. Colours represent combinations of low (L) (MIR < 3.81‰; access < 36.87%), middle (M) (MIR = 3.81–47.87‰; access = 36.87–42.17%), and high (H) (MIR ≥ 47.87‰; access ≥ 42.17%) quantile classes for both MIR and healthcare access.

# Supplementary tables

**Table S5.** Cross-validation of Bayesian geostatistical models of DHS indicators

| **Random CV** | **RMSE** | **MAE** | **R² (COD)** | **r² (corr.)** |
| --- | --- | --- | --- | --- |
| Access to basic sanitation service | 0.21 | 0.17 | 0.42 | 0.44 |
| Proportion of Fula | 0.23 | 0.16 | 0.52 | 0.54 |
| Stunting in children | 0.11 | 0.09 | 0.20 | 0.21 |
| Ownership of insecticide-treated nets | 0.14 | 0.11 | 0.45 | 0.46 |
| Literacy rate in women | 0.16 | 0.13 | 0.55 | 0.56 |
| **Temporal block CV** | **RMSE** | **MAE** | **R² (COD)** | **r² (corr.)** |
| Access to basic sanitation service | 0.21 | 0.17 | 0.40 | 0.44 |
| Proportion of Fula | 0.23 | 0.16 | 0.51 | 0.53 |
| Stunting in children | 0.11 | 0.09 | 0.20 | 0.21 |
| Ownership of insecticide-treated nets | 0.17 | 0.13 | 0.03 | 0.32 |
| Literacy rate in women | 0.16 | 0.13 | 0.56 | 0.58 |

*Note*. The MAE, RMSE, R² and r² values are the mean values calculated on the cross-validation test sets. The R² is the coefficient of determination (COD) and the r² is the square of the correlation between the observed and predicted values. Abbreviations: DHS (Demographic and Health Surveys), CV (cross-validation), MAE (mean absolute error), RMSE (root mean square error), COD (coefficient of determination), corr. (correlation).

**Table S6.** Cross-validation of Bayesian geostatistical models of access to healthcare

| **Access to healthcare** | **AUC** | **Accuracy** | **Recall** | **Precision** |
| --- | --- | --- | --- | --- |
| Random CV | 0.61 | 0.61 | 0.87 | 0.62 |
| Temporal block CV | 0.55 | 0.58 | 0.86 | 0.59 |

*Note*. Model performance was assessed through 5-repeated 5-fold cross-validation (CV) and temporal block CV by calculating the accuracy (proportion of correct predictions to all predictions), precision (proportion of true positives to all positive predictions), recall (proportion of true positives to all actual positives) and the Area Under Curve (AUC).

**Table S7.** Cross-validation metrics (MIR scale) for Bayesian geostatistical models of malaria incidence rates (MIR)

| **Random CV** | **RMSE** | **MAE** | **R² (COD)** | **r² (corr.)** |
| --- | --- | --- | --- | --- |
| Benchmark (BM) | 0.20 | 0.06 | 0.62 | 0.65 |
| BM for E1 | 0.47 | 0.29 | 0.40 | 0.48 |
| BM for E2 | 0.09 | 0.03 | 0.43 | 0.60 |
| BM for E3 | 0.04 | 0.01 | 0.26 | 0.29 |
| Wet season (WM) | 0.13 | 0.04 | 0.65 | 0.67 |
| Dry season (DM) | 0.08 | 0.02 | 0.54 | 0.56 |
| Endemicity 1 (E1) | 0.48 | 0.29 | 0.37 | 0.43 |
| Endemicity 2 (E2) | 0.08 | 0.02 | 0.57 | 0.69 |
| Endemicity 3 (E3) | 0.04 | 0.01 | 0.27 | 0.32 |
| **Temporal block CV** | **RMSE** | **MAE** | **R² (COD)** | **r² (corr.)** |
| Benchmark (BM) | 0.17 | 0.05 | 0.70 | 0.75 |
| BM for E1 | 0.41 | 0.25 | 0.55 | 0.65 |
| BM for E2 | 0.09 | 0.03 | 0.44 | 0.63 |
| BM for E3 | 0.04 | 0.01 | 0.19 | 0.30 |
| Wet season (WM) | 0.13 | 0.04 | 0.66 | 0.77 |
| Dry season (DM) | 0.07 | 0.02 | 0.63 | 0.66 |
| Endemicity 1 (E1) | 0.41 | 0.24 | 0.55 | 0.64 |
| Endemicity 2 (E2) | 0.08 | 0.03 | 0.49 | 0.68 |
| Endemicity 3 (E3) | 0.04 | 0.01 | 0.20 | 0.31 |

*Note*. The MAE, RMSE, R² and r² values are the mean values calculated on the cross-validation test sets using the exponentiated posterior means of log MIR. The R² is the coefficient of determination (COD) and the r² is the square of the correlation between the observed and predicted values. BM for E1, BM for E2 and BM for E3 refer to the performance of the benchmark model (BM) within endemicity groups 1, 2 and 3. Abbreviations: CV (cross-validation), MAE (mean absolute error), RMSE (root mean square error), COD (coefficient of determination), corr. (correlation).

**Table S8.** Cross-validation metrics (log scale) for Bayesian geostatistical models of malaria incidence rates (MIR)

| **Random CV** | **RMSE** | **MAE** | **R² (COD)** | **r² (corr.)** |
| --- | --- | --- | --- | --- |
| Benchmark (BM) | 1.06 | 0.83 | 0.74 | 0.74 |
| BM for E1 | 0.89 | 0.68 | 0.42 | 0.49 |
| BM for E2 | 1.09 | 0.87 | 0.56 | 0.57 |
| BM for E3 | 1.09 | 0.85 | 0.49 | 0.50 |
| Wet season (WM) | 1.03 | 0.81 | 0.75 | 0.75 |
| Dry season (DM) | 1.07 | 0.84 | 0.72 | 0.72 |
| Endemicity 1 (E1) | 0.85 | 0.64 | 0.48 | 0.52 |
| Endemicity 2 (E2) | 1.09 | 0.86 | 0.56 | 0.58 |
| Endemicity 3 (E3) | 1.07 | 0.84 | 0.51 | 0.52 |
| **Temporal block CV** | **RMSE** | **MAE** | **R² (COD)** | **r² (corr.)** |
| Benchmark (BM) | 1.04 | 0.81 | 0.75 | 0.76 |
| BM for E1 | 0.83 | 0.63 | 0.47 | 0.61 |
| BM for E2 | 1.11 | 0.87 | 0.54 | 0.58 |
| BM for E3 | 1.05 | 0.83 | 0.52 | 0.54 |
| Wet season (WM) | 1.02 | 0.80 | 0.75 | 0.76 |
| Dry season (DM) | 1.06 | 0.83 | 0.72 | 0.73 |
| Endemicity 1 (E1) | 0.78 | 0.59 | 0.54 | 0.63 |
| Endemicity 2 (E2) | 1.08 | 0.85 | 0.57 | 0.61 |
| Endemicity 3 (E3) | 1.05 | 0.83 | 0.52 | 0.55 |

*Note*. The MAE, RMSE, R² and r² values are the mean values calculated on the cross-validation test sets. The R² is the coefficient of determination (COD) and the r² is the square of the correlation between the observed and predicted values. BM for E1, BM for E2 and BM for E3 refer to the performance of the benchmark model (BM) within endemicity groups 1, 2 and 3. Abbreviations: CV (cross-validation), MAE (mean absolute error), RMSE (root mean square error), COD (coefficient of determination), corr. (correlation).

**Table S9.** Estimates of malaria cases and incidence rates for 2017–2021 by season and endemic group

| **Malaria cases** | **2017** | **2018** | **2019** | **2020** | **2021** |
| --- | --- | --- | --- | --- | --- |
| National level | 897,350 | 1,172,900 | 766,298 | 899,520 | 1,188,862 |
| Wet season | 589,953 | 808,109 | 429,836 | 603,786 | 666,002 |
| Dry season | 313,946 | 375,165 | 339,063 | 306,089 | 524,865 |
| Endemicity 1 group | 592,340 | 923,999 | 591,610 | 737,423 | 917,457 |
| Endemicity 2 group | 228,924 | 105,814 | 85,455 | 78,618 | 164,892 |
| Endemicity 3 group | 76,085 | 143,086 | 89,233 | 83,478 | 106,513 |
| **Malaria incidence (‰)** | **2017** | **2018** | **2019** | **2020** | **2021** |
| National level | 59.75 | 75.91 | 48.2 | 55.01 | 70.53 |
| Wet season | 39.28 | 52.3 | 27.04 | 36.93 | 39.51 |
| Dry season | 20.9 | 24.28 | 21.33 | 18.72 | 31.14 |
| Endemicity 1 group | 344.87 | 527.91 | 328.14 | 397.64 | 481.02 |
| Endemicity 2 group | 37.46 | 16.88 | 13.23 | 11.83 | 24.08 |
| Endemicity 3 group | 10.58 | 19.21 | 11.68 | 10.62 | 13.15 |

*Note*. Cases per health facility were first estimated by multiplying the observed malaria incidence rate (MIR) per health facility by the total potential catchment population (i.e. assuming 100% of the population sought health care for fever). For facilities with missing MIR data, we instead used the exponentiated Bayesian posterior mean log MIR from the relevant model instead (in order: BM, WM, DM, E1, E2, E3) to derive their estimated case counts. Cases were then summed and divided by the total population to calculate the annual MIR.

**Table S10.** Malaria incidence rates (MIR, per 1,000 people) for 2017–2021 per health district

| Region | Health district | 2017 | 2018 | 2019 | 2020 | 2021 |
| --- | --- | --- | --- | --- | --- | --- |
| Dakar | Dakar-Centre | 5.98 | 7.19 | 6.25 | 6.44 | 7.98 |
|  | Dakar-Nord | 14.62 | 17.6 | 21.04 | 11.21 | 19.9 |
|  | Dakar-Ouest | 7 | 5.36 | 7.81 | 4.27 | 6.6 |
|  | Dakar-Sud | 5.53 | 15.45 | 9.75 | 4.44 | 13.7 |
|  | Diamniadio | 18.74 | 12.96 | 19.2 | 19.04 | 17.88 |
|  | Guédiawaye | 9.53 | 6.12 | 7.4 | 4.61 | 6.56 |
|  | Keur Massar | 10.75 | 9.9 | 12.4 | 9.72 | 9.95 |
|  | Mbao | 5.29 | 3.8 | 3.13 | 3.31 | 3.98 |
|  | Pikine | 7.36 | 4.21 | 7.18 | 4.45 | 4.73 |
|  | Rufisque | 17.92 | 11.72 | 14.63 | 20.36 | 20.57 |
|  | Yeumbeul | 10.75 | 6.74 | 5.61 | 5.82 | 8.27 |
| Diourbel | Bambey | 4.63 | 5.5 | 5.22 | 3.62 | 4.69 |
|  | Diourbel | 138.26 | 23.05 | 14.95 | 8.87 | 21.41 |
|  | Mbacké | 26.74 | 10.63 | 5.62 | 9.08 | 13.14 |
|  | Touba | 221.17 | 85 | 71.1 | 65.89 | 170.7 |
| Fatick | Colobane | 5.38 | 6.61 | 2.41 | 2.33 | 3.77 |
|  | Dioffior | 3.32 | 2.18 | 1.26 | 1.33 | 2.01 |
|  | Foundiougne | 1.7 | 2.32 | 1.28 | 1.34 | 1.68 |
|  | Ndiob | 5.41 | 4.25 | 3.66 | 2.54 | 3.71 |
|  | Niakhar | 5.57 | 3.09 | 3.84 | 2.39 | 4.1 |
|  | Passy | 59.98 | 24.99 | 3.32 | 1.95 | 3.77 |
|  | Sokone | 25.57 | 7.07 | 1.74 | 3.29 | 4.78 |
| Kédougou | Kédougou | 797 | 885.37 | 755.39 | 708.24 | 957.1 |
|  | Salémata | 1077.31 | 1266.69 | 733.61 | 982.36 | 1112.98 |
|  | Saraya | 986.93 | 943.06 | 720.45 | 1098.4 | 1458.15 |
| Kaffrine | Birkelane | 14.93 | 18.14 | 9.15 | 6.49 | 9.48 |
|  | Kaffrine | 11.16 | 16.12 | 9.87 | 7.85 | 8.73 |
|  | Koungueul | 21.49 | 68.09 | 42.45 | 40.05 | 26.64 |
|  | Malem-Hodar | 12.17 | 29.09 | 17.5 | 17.43 | 15.09 |
| Kaolack | Guinguinéo | 7.28 | 5.58 | 2.85 | 2.8 | 3.11 |
|  | Kaolack | 113.22 | 59.86 | 24.36 | 20.05 | 76.58 |
|  | Ndoffane | 11.64 | 7.34 | 2.67 | 1.22 | 1.83 |
|  | Nioro du Rip | 7.02 | 5.61 | 3.57 | 4.32 | 5.11 |
| Kolda | Kolda | 274.52 | 425.72 | 251.69 | 353.08 | 477.99 |
|  | Médina Yoro Foulah | 214.57 | 439.34 | 203.15 | 274.54 | 282.07 |
|  | Vélingara | 418.57 | 544.52 | 400.78 | 412.73 | 502.21 |
| Louga | Coki | 6.79 | 4.99 | 7.39 | 3.51 | 6.52 |
|  | Dahra | 7.37 | 10.83 | 6.9 | 7.46 | 6.19 |
|  | Darou Mousty | 18.77 | 6.83 | 10.07 | 10.45 | 8.96 |
|  | Kébémer | 4.8 | 3.46 | 4.3 | 3.39 | 4.57 |
|  | Keur Momar Sarr | 1.66 | 2.15 | 2.13 | 1.77 | 2.3 |
|  | Linguère | 7.34 | 30.09 | 12.03 | 15.39 | 12.08 |
|  | Louga | 4.05 | 2.14 | 2.79 | 2.99 | 3.54 |
|  | Sakal | 2 | 1.31 | 1.18 | 1.63 | 1.64 |
| Matam | Kanel | 15.78 | 79.25 | 30.94 | 36.21 | 32.7 |
|  | Matam | 4.47 | 95.8 | 79.91 | 7.35 | 6.5 |
|  | Ranérou | 37.19 | 180.73 | 88.48 | 97.88 | 72.4 |
|  | Thilogne | 1.53 | 5.65 | 2.79 | 2.97 | 2.64 |
| Sédhiou | Bounkiling | 31.04 | 91.53 | 23.98 | 44.14 | 68.38 |
|  | Goudomp | 13.95 | 29.64 | 15.67 | 25.14 | 56.05 |
|  | Sédhiou | 33.29 | 64.8 | 25.4 | 44.25 | 69 |
| Saint-Louis | Dagana | 2.41 | 4.22 | 2.36 | 2.22 | 2.6 |
|  | Pete | 1.85 | 3.78 | 3.26 | 2.56 | 1.91 |
|  | Podor | 1.21 | 2.69 | 5.36 | 1.43 | 1.53 |
|  | Richard Toll | 1.14 | 1.12 | 1.22 | 1.26 | 1.67 |
|  | Saint-Louis | 2.88 | 2.63 | 1.95 | 1.83 | 2.71 |
| Tambacounda | Bakel | 74.81 | 169.69 | 91.31 | 129.6 | 126.4 |
|  | Dianké Makha | 718.15 | 971.24 | 531.88 | 830.72 | 1020.21 |
|  | Goudiry | 307.5 | 533.95 | 268.46 | 367.72 | 454.25 |
|  | Kidira | 370.38 | 505.47 | 212.72 | 308.15 | 393.34 |
|  | Koumpentoum | 28.25 | 264.9 | 122.44 | 114.16 | 99.67 |
|  | Makacoulibantang | 186.28 | 580.55 | 339.9 | 244.89 | 142.94 |
|  | Tambacounda | 282.97 | 531.81 | 317.47 | 411.01 | 472.4 |
| Thiès | Joal | 10.89 | 6.03 | 3.55 | 4.68 | 5.52 |
|  | Khombole | 5.65 | 5.41 | 7.76 | 6.03 | 9.34 |
|  | Mbour | 6.37 | 5.97 | 7.16 | 4.83 | 6.37 |
|  | Mékhé | 13.14 | 6.58 | 8.46 | 9.31 | 11.31 |
|  | Popenguine | 3.59 | 3.46 | 5.58 | 3.77 | 6.44 |
|  | Pout | 10.72 | 6.28 | 9.19 | 7.92 | 9.74 |
|  | Thiadiaye | 11.73 | 7.72 | 3.95 | 4.15 | 4.03 |
|  | Thiès | 3.37 | 7.87 | 4.92 | 2.03 | 2.72 |
|  | Tivaouane | 8.59 | 3.99 | 4.51 | 5.05 | 4.22 |
| Ziguinchor | Bignona | 14.62 | 11.34 | 12.22 | 17.02 | 26.21 |
|  | Diouloulou | 9.91 | 5.96 | 6.03 | 5.96 | 10.66 |
|  | Oussouye | 8.24 | 7.73 | 4.92 | 2.94 | 5.13 |
|  | Thionck Essyl | 6.94 | 5.85 | 3.78 | 4.07 | 8.86 |
|  | Ziguinchor | 18.55 | 13.24 | 14.63 | 16 | 30.14 |

*Note.* Cases per health facility were first estimated by multiplying the observed malaria incidence rate (MIR) per health facility by the total potential catchment population (i.e. assuming 100% of the population sought health care for fever). For facilities with missing MIR data, we instead used the exponentiated Bayesian posterior mean log MIR from the benchmark model (BM) to derive their estimated case counts. Cases were then summed per health district and divided by the total district population to calculate the MIR.

**Table S11.** Malaria interventions implemented between 2017 and 2021 in Senegal

| **Year** | **Intervention** | **Region** |
| --- | --- | --- |
| 2017 | SMC | KKT, Sédhiou |
|  | IRS | 4 districts: Malem Hoddar and Koungheul (Kaffrine), Koumpentoum (Tambacounda) and Nioro (Kaolack) |
|  | PECADOM Plus | 40 districts |
|  | Case investigation | 10 districts in North (Saint-Louis, Matam) |
|  | Routine ITN distribution | Health and community channels. |
| 2018 | PECADOM Plus | 35 districts |
|  | Case investigation | 10 districts in North (Saint-Louis, Matam) |
|  | Routine ITN distribution | Health and community channels. |
| 2019 | SMC | KKT, Sédhiou, Diourbel, Kaolack |
|  | IRS | 2 districts: Ranérou and Kanel (Matam region) |
|  | PECADOM Plus | 18 districts |
|  | Case investigation | 10 districts in North (Saint-Louis, Matam) |
|  | Routine ITN distribution | Health and community channels. |
|  | Mass ITN distribution | National coverage |
| 2020 | SMC | KKT, Sédhiou, Kaolack |
|  | IRS | 8 districts in North (Matam, Louga) and South (Kédougou, Tambacounda, Kaffrine) |
|  | PECADOM Plus | 35 districts |
|  | Case investigation | 10 districts in North (Saint-Louis, Matam) |
|  | Routine ITN distribution | Health, community and school channels. |
| 2021 | SMC | KKT, Sédhiou, Kaolack |
|  | IRS | 8 districts in North (Matam, Louga) and South (Kédougou, Tambacounda, Kaffrine) |
|  | PECADOM Plus | 16 districts |
|  | Case investigation | 37 districts |
|  | Routine ITN distribution | Health, community and school channels. |

*Note.* SMC (seasonal malaria chemoprevention) refers to the distribution of antimalarial drugs to children under five during the transmission season. IRS (indoor residual spraying) is the spraying of insecticides inside houses to reduce malaria transmission. PECADOM Plus involves weekly door-to-door visits for malaria diagnosis and treatment of suspected cases. Case investigation requires prompt investigation of each reported case and targeted drug administration. ITN (insecticide-treated net) distribution is the provision of ITNs to households to prevent malaria infection, either through mass campaigns or routinely through health systems, schools and communities.

# References

[1]	Cairns ME, Walker PGT, Okell LC, et al. Seasonality in malaria transmission: implications for case-management with long-acting artemisinin combination therapy in sub-Saharan Africa. Malaria Journal. 2015;14(1):321.

[2]	Ndione B. Migration au Sénégal: profil national 2018 [Internet]. Sénégal: Agence Nationale de la Statistique et de la Démographie du Sénégal (ANSD); 2018. Available from: https://publications.iom.int/system/files/pdf/mp_senegal_2018_fr.pdf.

[3]	World Cities Database [Internet]. simplemaps. Interactive Maps & Data. 2025. Available from: https://simplemaps.com/data/world-cities.
