# Supplementary material for: Where and why malaria persists in heterogeneous transmission settings: insights from routine health facility data
Source: BMJ Glob Health. 2026 Aug 7;11(8):e023003. doi: 10.1136/bmjgh-2025-023003 (PMC13475453; doi:10.1136/bmjgh-2025-023003)
Supplement: Supplementary file 3 [file bmjgh-11-8-s003.docx]

### BMJ Global Health Author Reflexivity Statement

Adapted from Morton, B., Vercueil, A., Masekela, R., Heinz, E., Reimer, L., Saleh, S., Kalinga, C., Seekles, M., Biccard, B., Chakaya, J., Abimbola, S., Obasi, A. and Oriyo, N. (2022), Consensus statement on measures to promote equitable authorship in the publication of research from international partnerships. Anaesthesia, 77: 264-276. <https://doi.org/10.1111/anae.15597>

| **Study conceptualisation** | |
| --- | --- |
| 1. How does this study address local research and policy priorities? | Malaria is a major public health issue in Senegal and a policy priority given the 2030 elimination target. |
| 2. How were local researchers involved in study design? | All authors were involved in the study design by contributing to the formulation of research questions and providing input on the interpretation of data and results. |
| **Research management** | |
| 3. How has funding been used to support the local research team(s)? | The project led to mobility opportunities for researchers from the Université Cheikh Anta Diop (Senegal) and the University of Namur (Belgium) and supported the organization of a workshop aimed at building local capacity in analytical methods. |
| **Data acquisition and analysis** | |
| 4. How are research staff who conducted data collection acknowledged? | Not applicable. The study did not include data collection activities. |
| 5. How have members of the research partnership been provided with access to study data? | The authors were given access to DHIS2 data, which had been aggregated at the health facility level, from the Laboratoire Dynamiques Territoriales et Santé at the Université Cheikh Anta Diop de Dakar in Senegal. |
| 6. How were data used to develop analytical skills within the partnership? | The project included a workshop at the University Cheikh Anta Diop (Dakar) during the *Journées de la Géographie de la Santé* in November 2024, aimed at local researchers and master students, to transfer analytical methods and develop their data analysis skills. |
| **Data interpretation** | |
| 7. How have research partners collaborated in interpreting study data? | All research partners (and therefore authors) that took part in this study have participated in results and data interpretation through multiple meetings/discussions. |
| **Drafting and revising for intellectual content** | |
| 8. How were research partners supported to develop writing skills? | Research partners were supported through active participation in reviewing the manuscript. |
| 9. How will research products be shared to address local needs? | Research products have been and will be shared with local stakeholders through workshops and presentations. |
| **Authorship** | |
| 10. How is the leadership, contribution and ownership of this work by LMIC researchers recognised within the authorship? | All researchers that contributed to the manuscript and study are recognised as authors. |
| 11. How have early career researchers across the partnership been included within the authorship team? | Early career researchers from all partner countries were included as authors when they contributed to the study/paper. |
| 12. How has gender balance been addressed within the authorship? | Gender balance has been addressed by ensuring a diverse authorship team composed of four women and two men. |
| **Training** | |
| 13. How has the project contributed to training of LMIC researchers? | The project contributed to the training of LMIC researchers by organizing a workshop at the University Cheikh Anta Diop (Dakar) during the *Journées de la Géographie de la Santé* in November 2024. The workshop targeted local researchers and master’s students and focused on transferring the study methods. |
| **Infrastructure** | |
| 14. How has the project contributed to improvements in local infrastructure? | Not applicable. |
| **Governance** | |
| 15. What safeguarding procedures were used to protect local study participants and researchers? | The project did not involve primary data collection or direct interaction with participants. All data used were already available anonymized and aggregated (therefore not available at individual level), eliminating risks to both participants and researchers. |
